# Riemannian metric learning for alignment of spatial multiomics

**DOI:** 10.1093/bioinformatics/btag220

**Published:** 2026-07-07

**Authors:** Peter Halmos, Yufan Xia, Benjamin J Raphael

**Affiliations:** Department of Computer Science, Princeton University, Princeton, NJ 08544, United States; Department of Computer Science, Princeton University, Princeton, NJ 08544, United States; Department of Computer Science, Princeton University, Princeton, NJ 08544, United States

## Abstract

**Motivation:**

Recent spatial technologies measure the transcriptome, epigenome, proteome, metabolome, and other modalities from thousands of cells across a tissue. Most assays typically profile only one modality from a tissue slice, raising the question of how to align spatial data from heterogeneous feature spaces. While multiple approaches have been developed for multi-modal integration of single-cell datasets, few existing techniques perform spatial alignment across arbitrary modalities incorporating both spatial and feature information.

**Results:**

We introduce Manifold Gromov-Wasserstein (MGW), a metric-learning framework that exploits the product structure of spatial multiomics to infer modality-specific Riemannian *pull-back* metrics with neural fields. MGW aligns Riemannian distances induced by these metrics via Gromov-Wasserstein optimal transport, yielding a hyperparameter-free cost across arbitrary modalities sharing a spatial base. The formulation enjoys theoretical invariances—including orthogonal transformations of the spatial and feature domains as well as global feature scalings. We demonstrate the advantages of MGW on multiple alignment tasks, including Stereo-Seq spatiotemporal transcriptomics of mouse embryo, Xenium and Visium spatial transcriptomics of colorectal cancer, and spatial metabolomics-transcriptomics from human striatum and kidney cancer. MGW recovers biologically meaningful correspondences and spatially coherent tissue structures, outperforming existing OT and non-OT based multi-modal baselines.

**Availability and implementation:**

Software is available at https://github.com/raphael-group/MGW.

## 1 Introduction

Spatially-resolved transcriptomic (SRT) technologies (Ståhl et al. 2016, Rao et al. 2021, Moses and Pachter 2022) are a seminal advance toward a comprehensive understanding of spatial biology (Baccin et al. 2020, Ji et al. 2020), but they measure only one of the many biological modalities which define the state of a cell. Recent advances in spatial omics have expanded measurement capabilities beyond the transcriptome, enabling profiling of the metabolome, epigenome, and proteome. While the majority of these technologies only measure a single modality for a given slice, emerging experimental technologies combine measurements of the transcriptome with profiling of the epigenome in spatial ATAC-RNA-Seq and spatial CUT & Tag-RNA-Seq (Zhang et al. 2023), profiling of spatial metabolomics and histology (Vicari et al. 2024), profiling of open chromatin and T-cell receptor sequences (Russell et al. 2024), and profiling of protein markers (Ben-Chetrit et al. 2023). To transform this unprecedented, heterogeneous spectrum of spatial data into comprehensive multiomic maps, computational integration across datasets with differing modalities is essential. While modern computational techniques have offered great promise for understanding biology from a functional lens—from cell-differentiation dynamics (Schiebinger et al. 2019, Sha et al. 2024, Halmos et al. 2025a) to spatial heterogeneity (Hu et al. 2021, Dong and Zhang 2022, Xu et al. 2022, Tanevski et al. 2025) and cellular niches (Fischer et al. 2023, Haviv et al. 2024)—extending these insights to the multiomic universe requires *alignment* across heterogeneous modalities.

Multiple works have tackled the problem of aligning spatial transcriptomics slices (Zeira et al. 2022, Clifton et al. 2023, Liu et al. 2023, Halmos et al. 2025b, Klein et al. 2025). Techniques for spatial alignment have proven highly effective, but face the challenge of how to weight similarity between the transcriptomic features at each spatial location with similarity between the spatial geometry of the tissue slices. Some methods such as STAlign (Clifton et al. 2023) avoid this challenge by relying on spatial information alone. In contrast, PASTE (Zeira et al. 2022) introduced the application of *fused Gromov-Wasserstein* (FGW) optimal transport (Titouan et al. 2019) to spatial alignment, which includes a hyperparameter α that describes the trade-off between spatial and feature similarity. FGW was adopted by multiple subsequent works including PASTE2 for partial alignment (Liu et al. 2023), DeST-OT for semi-relaxed alignment (Halmos et al. 2025b), moscot for unbalanced alignment (Klein et al. 2025), and the learning of cell-fate landscapes (Huizing et al. 2025). However, the hyperparameter in FGW is often difficult to set properly and all of the noted approaches work with only a single modality, usually transcriptomics.

Alignment of single-cell datasets from different modalities is also a well-studied problem. Multimodal alignment presents a unique challenge, as these spaces may have differing dimensions, incomparable scales and geometries, and no common basis of coordinates on which points can be compared. However, aligning across heterogeneous spaces is the most general and powerful form of alignment: it subsumes any notion of mode, from experimental batch to time point to technology. Works such as SCOT (Demetci et al. 2022b), SCOTv2 (Demetci et al. 2022a), and moscot (Klein et al. 2025) align single-cell datasets across modalities using Gromov-Wasserstein optimal transport, matching the modality-spaces based on their pairwise distance structures. In contrast, numerous non-OT approaches—based on variational autoencoders, nearest-neighbor anchoring, or matrix-factorization (Stuart et al. 2019, Welch et al. 2019, Hao et al. 2021, Litinetskaya et al. 2025)—perform multiomic integration by learning a shared latent representation, but typically do not return explicit cross-dataset alignments. A universal limitation of existing OT-based multimodal alignment methods is that they do not leverage spatial information and do not *learn* a cost function. Instead, they rely on Euclidean or graph distances computed symmetrically within each modality on the raw features.

One approach to spatial multi-modal integration is to learn joint embeddings of spatial multiomics via graph representations or autoencoders, as in MultiGate (Miao et al. 2025) and SpatialMeta (Tian et al. 2025) but these methods presume *pre-aligned* cells. Even when an alignment module is included for spatial multiomics [e.g. in Tian et al. (2025)], registration operates on *coordinates*, and not on the feature-space geometry. Another recent and parallel line of work has investigated learning of the *Riemannian metric* of the data-manifold (Arvanitidis et al. 2022), with applications to learning the latent manifold with autoencoders (Sun et al. 2024, Palma et al. 2025). However, no existing techniques have bridged modality-specific metric learning with spatial alignment across heterogeneous modalities.

*Contributions.* We introduce *Manifold Gromov-Wasserstein* (MGW), an algorithm to align spatial omics datasets across arbitrary modalities. The key insight of MGW is to learn a cost whose mapping across modality spaces preserves the *intrinsic manifold structure* of each space. MGW is based on the observation that a spatial omics dataset can be viewed as a function from physical (Euclidean) space $E\subset R^{2}$ or $R^{3}$ to a high-dimensional feature, or modality, space M. Since each modality shares the same base space *E*, MGW utilizes a standard approach in Riemannian geometry for studying different manifolds sharing a common base: the *Riemannian pull-back metric*, which pulls the geometry of the modality spaces back onto the common Euclidean space. To compute the pull-back metric, we represent each spatial-omic dataset with a *neural field*: an implicit map from physical space *E* into the respective modality space. Finally, MGW computes an alignment using Gromov-Wasserstein Optimal Transport on the Riemannian geodesic distances in each modality. Thus, MGW offers a geometrically unified technique for spatial multimodal alignment and is free of hyperparameters for weighting between spaces.

We show that MGW is a natural measure of distance between multi-modal data, with invariance to rigid-body transformations of the spatial coordinates and invariance to orthogonal transformations, offsets, and homogeneous scalings of the features. Additionally, MGW recovers GW on spatial coordinates when the fields are identity maps. We demonstrate that MGW outperforms existing spatial alignment methods across four different spatial-omics alignment tasks spanning five measurement technologies. MGW bridges neural representation and feature-learning with optimal transport, offering a natural way to *learn* costs for optimal transport to align across different modalities.

## 2 Materials and methods

A spatial-omics dataset $D={\left(x^{i}\right)}_{i=1}^{n}$ is a set of pairs $x^{i}=(s^{i},X^{i})$, where $s^{i}$ is a spatial location and $X^{i}$ is a feature measured at the location (such as a transcript count vector). For spatial multiomics alignment one has a common Euclidean base space *E* (e.g. $E=R^{2},R^{3}$), and a pair of spaces $M\subset R^{d},N\subset R^{p}$ representing the space for each measured modality (e.g. M is transcriptomics, and N is metabolomics). Let $\{x^{1},\ldots ,x^{n}\}\subset E\times M$ and $\{y^{1},\ldots ,y^{m}\}\subset E\times N$ be two such datasets, and let $\Delta_{k}$ be the probability simplex of size *k*. We encode the two datasets as probability measures $\mu =\sum_{i}a_{i}\delta_{x^{i}}$ and $\nu =\sum_{j}b_{j}\delta_{y^{j}}$ with probability vectors $a\in \Delta_{n},b\in \Delta_{m}$ representing the weight of each point (e.g. uniform). We define a coupling between a and b to be a matrix $\pi \in R_{+}^{n\times m}$ with marginals $\pi 1_{m}=a$ and $\pi^{⊤}1_{n}=b$. The set of all couplings between a and b is denoted by $\Pi_{a,b}$. We begin by introducing the general problem of aligning such multimodal data.

Problem 1 (Multimodal Spatial Alignment.)

*Given spatial datasets* ${\left(s^{i},X^{i}\right)}_{i=1}^{n}\subset E\times M$*and* ${\left(t^{j},Z^{j}\right)}_{j=1}^{m}\subset E\times N$*with modality spaces* M*and* N*over a common Euclidean base E, and a cross-space cost* $C_{M\times N}:[E\times M]\times [E\times N]\to R,$*find a coupling* $\pi^{⋆}$*between the modalities, which minimizes the expected cost*

(1) $$\underset{\pi \;\in \;\Pi_{a,b}}{\min}E_{(i,j)∼\;\pi }C_{M\times N}\left(\left(s^{i},X^{i}),(t^{j},Z^{j}\right)\right).$$

For fixed cost $C_{M\times N}(\cdot ,\cdot )$, Problem 1 is well-defined and the best alignment can be found using optimal transport (OT) [See (7) and (Alignment with Manifold Gromov-Wasserstein)]. The true challenge, then, is in determining or learning the most appropriate notion of cost. As different modality spaces exhibit incomparable dimensions, scalings, and geometries, identifying such a cost is difficult. A standard approach in spatial omics is the Fused Gromov-Wasserstein (FGW) cost, a convex combination of cross-dataset feature distances and within-dataset spatial distances (Titouan et al. 2019, Zeira et al. 2022). This poses existential limitations for Problem 1: FGW requires direct feature comparisons, which are undefined across dimensionally incompatible modalities.

The key principle behind MGW is to not pre-define a cost, but to instead *learn* a cost which preserves the *intrinsic manifold structure* of each modality space. This is not only to map across heterogeneous spaces, but also to preserve non-linear manifold geometry. Even when two datasets exist in spaces which are not directly comparable, the two modalities may exhibit a common geometry when viewed relatively within each space. To define such a cost, we make two key modeling assumptions:

- **(A1)** The modality spaces can be modeled by *Riemannian manifolds* (M,g) and (N,h) equipped with associated Riemannian metrics to measure distances.
- **(A2)** A pair of smooth mappings exist from the base Euclidean space into the respective modality spaces. We denote these by φ:E→M and ψ:E→N.

We note (A1) automatically holds for the base Euclidean space $\left(E,g^{E}\right)$ with metric being the identity $g^{E}=\mathrm{id}$. Given (A1) and (A2), the core question of our work then becomes the following:

*Can we learn a cost between arbitrary modalities measured over Euclidean space which can preserve the intrinsic geometry of the underlying data manifolds?*

### 2.1 Manifold-Pullback and pullback metric

We describe how MGW learns modality-specific notions of distance and pulls them back to the underlying Euclidean space. To make this precise, we first recall a few preliminaries from Riemannian geometry. Given a smooth manifold *M*, a *Riemannian metric* or *metric tensor* assigns to each point p∈M a symmetric, positive-definite bilinear form $g_{p}:T_{p}M\times T_{p}M\to R$ on the tangent space $T_{p}M$ such that the coefficients $g_{ij}(p)$ vary smoothly in every local coordinate chart. The metric acts as an inner product on tangent vectors in $T_{p}M$ and defines a local notion of length and angle. When $M=R^{d}$, the canonical choice g(X,Y):=〈X,Y〉 yields the standard Euclidean geometry. The pair (M,g) is called a *Riemannian manifold*.

**Figure 1:**
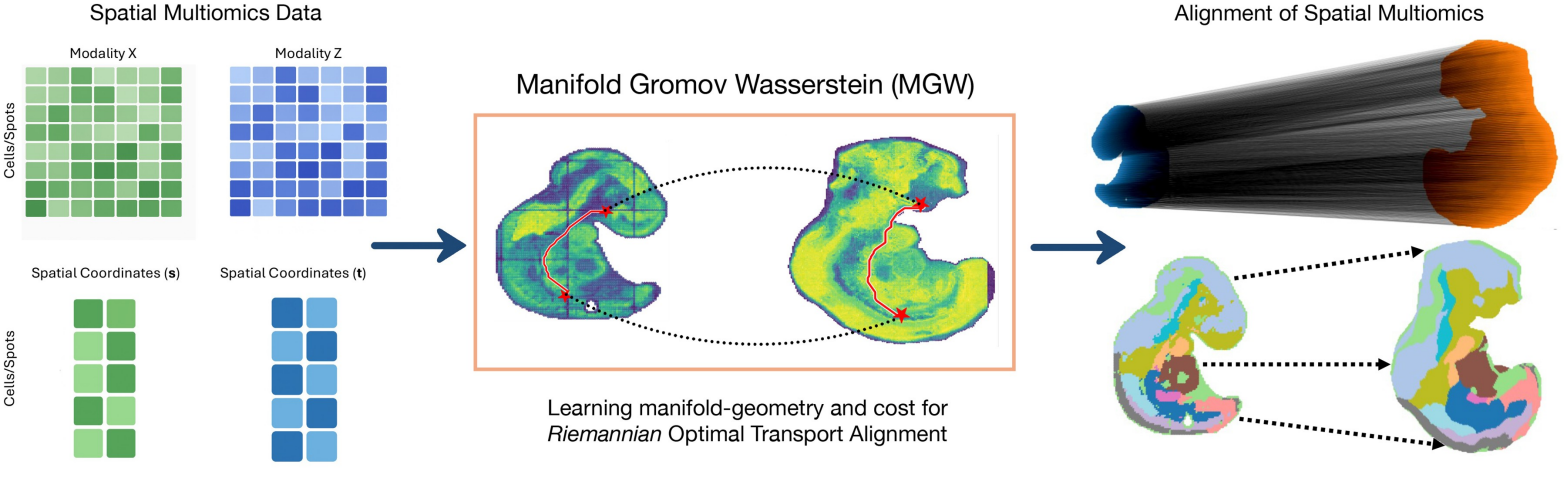
Overview of Manifold Gromov-Wasserstein (MGW). (Left) Two spatial multiomic datasets are input including both features for each modality and spatial coordinates. (Middle) MGW learns the intrinsic Riemannian geometry of each dataset with neural fields and aligns Riemannian distances. (Right) The alignment yields a non-linear mapping between cells measured in arbitrary modalities while preserving the intrinsic geometry of each dataset.

To extend these objects to spatial-multiomics, we need to learn the geometry of the features over physical space. We note that a spatial dataset $D={\left(s^{i},X^{i}\right)}_{i=1}^{n}$ can be viewed as *n* samples ${\left(s^{i},\varphi (s^{i})=X^{i}\right)}_{i=1}^{n}$ of tissue coordinates and a function φ:E→M mapping the tissue coordinates (Euclidean space) into the modality space M. We represent this function for each modality using an *implicit neural field* φ:E→M,ψ:E→N. Fields offer a different perspective on spatial multiomics: rather than representing the data as a finite grid or matrix of spatial and feature coordinates (Fig. 1), one views the data *implicitly* as a continuous field of features over physical space. Spatial multiomics data are well-suited to field-based parametrizations: gene expression or other molecular modalities vary smoothly and continuously across tissue (Chitra et al. 2025), and exhibit non-linear *isodepth* coordinates along which expression changes with a differentiable gradient. For MGW, the key value of the field-based representation is in capturing the spatial differential structure underlying modality variation over space. With access to spatial derivatives $\partial_{x}^{k}\varphi_{\theta }$, one can access essential geometric objects—gradients, curvature, and induced metric tensors.

Following (Chitra et al. 2025), we learn these fields as a coordinate-to-feature regression minimizing the mean-squared loss


$$\underset{\theta }{\min}\frac{1}{2n}\sum_{i=1}^{n}||\varphi_{\theta }(s^{i})-X^{i}|{|}_{2}^{2},\;\text{and}\;\underset{\eta }{\min}\frac{1}{2m}\sum_{j=1}^{m}||\psi_{\eta }(t^{j})-Z^{j}|{|}_{2}^{2}.$$

And taking $\psi :=\psi_{\eta },\varphi :=\varphi_{\theta }$ to be the implicit representations of each omics slice defined as a field defined over Euclidean space. For our architecture, we restrict to $C^{\infty }$ activations; in particular, we use the SiLU function. This ensures that $\psi_{\eta },\varphi_{\theta }$ have smooth Jacobians and represent smooth submanifolds, when viewed as local parametrizations mapping from Euclidean space *E* into the respective modalities. Many Jacobians in neural representations of omics data play key roles: gene-over-gene Jacobians characterize temporal gene-regulatory networks (Sha et al. 2024) and gene-over-space or modality-over-space Jacobians (Definition 1) characterize the gradients and curvature of a modality over physical space (Ma et al. 2022, Chitra et al. 2025).

While biological tissues may exhibit sharp boundaries (e.g. in cell-type or tumor-stroma boundaries), the smoothness of φ,ψ is a standard modeling assumption in implicit neural representations which ensures differentiability. In this framework, sharp discontinuities are effectively modeled by inferring a high gradient norm, which translates into a high distance for crossing distinct biological boundaries. As the modality-over-space Jacobian is central to this work, we recall it below.

Definition 1 (Space-Modality Jacobian.)

*Let* φ:E→M*denote a field mapping spatial coordinates* $s\in E\subset R^{d_{E}}$*to a feature-space* $M\subset R^{d}$*. At each point s, the space-modality Jacobian is the differential*

(2) $$J_{\varphi }(s)={\left[\frac{\partial }{\partial s_{j}}\varphi^{i}(s)\right]}_{ij}$$

(2) encodes how spatial displacements change modality (feature) abundances. As *s* varies over *E*, $J_{\varphi }(\cdot ):E\to L(TE,T_{\varphi }M)$ defines a smooth *Jacobian tensor field* of order (1,1) on *E*. In other words, each point *s* in *E* maps to a Jacobian encoding the manner in which spatial displacements change features at that point. Now, given the Jacobian (2), we define the *pullback metric* of the fields φ,ψ onto the Euclidean base *E*.

Definition 2 (Modality Pullback Metric).

*Given a field* φ*with Jacobian field* $J_{\varphi }(\cdot ):E\to L(TE,T_{\varphi }M)$*, at a point x the Pullback-Metric induced by* φ*for modality M is*

(3) $$g^{M}(x)=J_{\varphi }{\left(x\right)}^{⊤}J_{\varphi }(x)$$

A symmetric, positive-definite (0,2) tensor on the tangent space $T_{x}E$. The associated inner product is given by

(4) $$g^{M}(x)(u,v):=\langle J_{\varphi }(x)u,J_{\varphi }(x)v\rangle$$

The Riemannian pull-back metric is a classical approach in differential geometry for comparing manifolds in different spaces which are maps from a common space. Given our pair of smooth neural fields mapping from Euclidean space into the two modality spaces, each mapping implicitly defines a Riemannian pull-back metric on the base space through the Jacobians $J_{\varphi }$ and $J_{\psi }$.

While we offer a more formal description of this pull-back metric in Section 1.1, this metric has a simple interpretation. Intuitively, $g^{M}(x)$ (3) encodes the local anisotropy of modality/feature variation along different spatial directions in the modality *M*. A pair of points is deemed close if small spatial perturbations induce similar changes in the modality field. Thus, it “pulls” modality-specific structure back on the common Euclidean space. By mapping from Euclidean space into the modality spaces we are allowed to work in reference to the Euclidean space common to two spatial multiomics datasets and compare distances in this common space.

### 2.2 Riemannian geodesics in the pullback metric

Given a Riemannian manifold (M,g), the *Riemannian (geodesic) distance* d(p,q) between a pair p,q∈M is the length of the shortest path γ:[0,1]→M connecting them, defined as


(5) $$d(p,q):=\underset{\begin{array}{c} \gamma \in C^{\infty }[0,1]\\ \gamma (0)=p,\;\gamma (1)=q \end{array}}{\inf}\left\{\int_{0}^{1}\sqrt{g(\gamma (s))(\dot{\gamma }(s),\dot{\gamma }(s))}ds\right\}$$

This coincides with the Euclidean distance when *M* is flat, and captures shortest paths in space curved according to the metric *g*. Now, given the pair of pull-back metrics for the two modalities defined on the common Euclidean space *E*


$$g^{M}(p)=J_{\varphi }{\left(p\right)}^{⊤}J_{\varphi }(p),\;g^{N}(q)=J_{\psi }{\left(q\right)}^{⊤}J_{\psi }(q)$$

**Figure 2:**
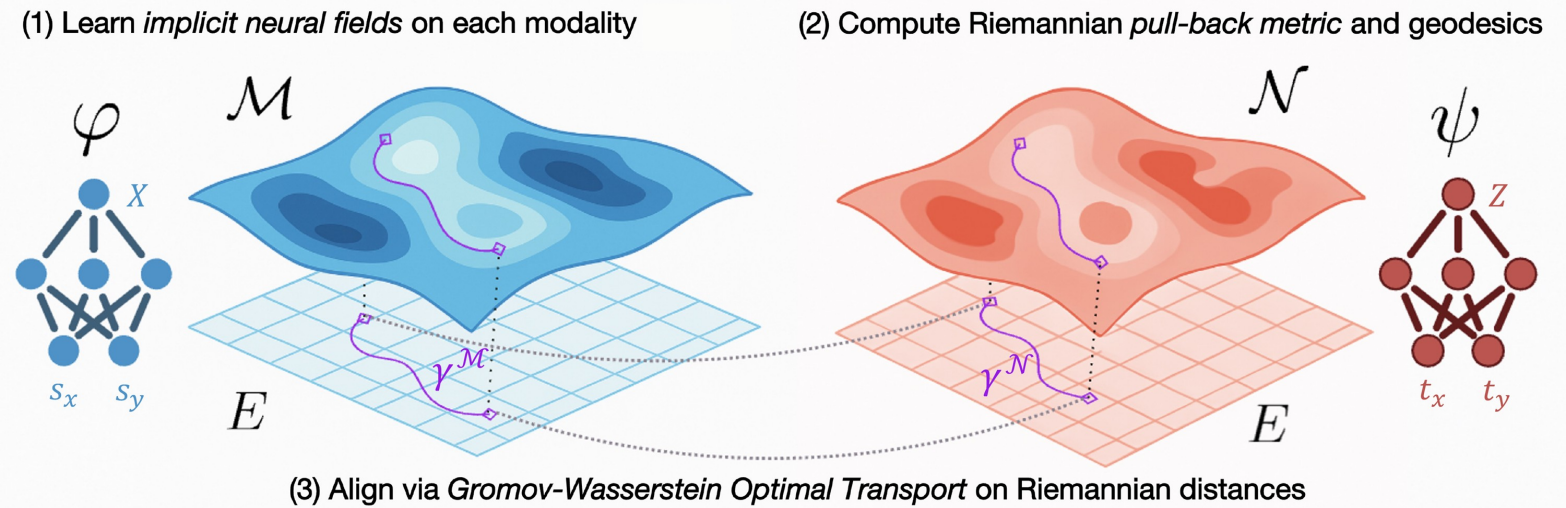
Manifold Gromov-Wasserstein (MGW). φ,ψ map from a Euclidean space *E* to distinct modality spaces M and N. The maps φ,ψ define Riemannian pull-back metrics $g^{M}$ and $g^{N}$ through their Jacobians, from which one can compute geodesics $\gamma^{M},\gamma^{N}$. Gromov-Wasserstein optimal transport yields an optimal coupling with respect to the Riemannian geodesic distances.

One may define Riemannian distances with respect to the pull-backs $g^{M}$ and $g^{N}$ for each modality (Definition 2). In particular, for each pair of spatial points $s,s^{\prime }\in E$ in our first dataset and $t,t^{\prime }\in E$ in our second, we compute geodesics $\gamma^{M}$, $\gamma^{N}$ in the modality-specific metrics $g^{M}$, $g^{N}$ and their Riemannian distance:


(6) $$d^{M}(s,s^{\prime }):=\underset{\begin{array}{c} \gamma^{M}\;\in \;C^{\infty }[0,1]\\ \gamma^{M}(0)=s,\;\gamma^{M}(1)=s^{\prime } \end{array}}{\inf}\left\{\int_{0}^{1}\sqrt{g^{M}(\gamma )(\dot{\gamma }(s),\dot{\gamma }(s))}ds\right\},$$

and resp. $d^{N}(t,t^{\prime })$. These paths $\gamma^{M}$ and $\gamma^{N}$ are intuitive, corresponding to a pull-back of the geodesics on each modality space onto *E* through the special requirement that φ(E)=M and ψ(E)=N (Fig. 2). Riemannian distances are well-suited to preserving sharp boundaries in biological data: crossing a boundary causes $|J_{\varphi }(s){|}^{2}$ and thus $|g^{M}{|}_{F}^{2}$ to increase, so that one incurs a major penalty for crossing a biological boundary or discontinuity.

Following training of the neural fields φ,ψ, computation of $g^{M}$ and $g^{N}$ at each point relies evaluation of the Jacobian, which is easily accomplished with automatic differentiation. However, (6) requires computing continuous, shortest-path geodesic curves. To approximate these, we simply discretize these paths at the resolution of the underlying spatial coordinate grid. Specifically, given these metrics the geodesics are approximated by building a sparse *k*-NN graph $G_{M}$ on the spatial points $\{s^{i}\}\subset E$ with weights given by the local arc-length in the Riemannian metric between each neighboring pair of points. For edge $\left(i,i^{\prime }\right)$ one may define $\Delta_{ii^{\prime }}=(s^{i}-s^{i^{\prime }})$ and compute the local, symmetrized arc-length as


$$w_{M}^{ii^{\prime }}=\frac{1}{2}\sqrt{\Delta_{ii^{\prime }}^{⊤}g_{i}^{M}\Delta_{ii^{\prime }}}+\frac{1}{2}\sqrt{\Delta_{ii^{\prime }}^{⊤}g_{i^{\prime }}^{M}\Delta_{ii^{\prime }}}.$$

$\Delta_{ii^{\prime }}^{⊤}g_{i}^{M}\Delta_{ii^{\prime }}$ can be viewed as a per-node Mahalanobis distance. One repeats this for the second modality space N and constructs $w_{N}^{jj^{\prime }}$ with graph $G_{N}$ on the spatial coordinates $\left\{t^{j}\right\}$ of the second dataset. Now, with both spatial coordinates (vertices) and arc-lengths in the metric (edge weights), the Riemannian geodesic distances (6) are easily computed with efficient routines for all-pairs shortest paths (APSP) on a sparse graph. Graph-based approximation of Riemannian geodesics on a *k*-NN graph is standard in manifold-learning and geometric graph methods, and scales well to typical spatial omics resolutions. We perform a sensitivity analysis on the *k*-NN parameter *k* in Section 5.4, available as supplementary data at *Bioinformatics* online (Fig. 7, available as supplementary data at *Bioinformatics* online, Table 5, available as supplementary data at *Bioinformatics* online).

### 2.3 Alignment with manifold Gromov-Wasserstein

We begin by recalling the Wasserstein formulation of optimal transport. Given a cost matrix of distances between the points $X^{i},Y^{j}\in X$, $C_{ij}:=c(X^{i},Y^{j})$, Wasserstein optimal transport seeks a coupling $\pi \in \Pi_{a,b}$ of minimal cost with respect to *c*


(7) $$\begin{array}{c} W_{c}(\mu ,\nu ):=\underset{\pi \in \Pi_{a,b}}{\min}{\langle C,\pi \rangle }_{F}. \end{array}$$

Where ${\langle A,B\rangle }_{F}=\sum_{ij}A_{ij}B_{ij}$ denotes the Frobenius (matrix) inner product. While the Wasserstein formulation finds a coupling minimizing the distances between two points in a common space, often one seeks to compare $\{X^{1},\ldots ,X^{n}\}\subset X$ and $\{Y^{1},\ldots ,Y^{m}\}\subset Y$ in distinct metric spaces X,Y. *Gromov-Wasserstein* optimal transport (Mémoli 2011) addresses this by allowing two cost functions $c_{1}:X\times X\to R_{+}$ and $c_{2}:Y\times Y\to R_{+}$, and instead comparing the metric distortion under π


$$\begin{array}{c} {\text{GW}}_{\mu ,\nu }=\underset{\pi \in \Pi_{a,b}}{\min}\sum_{ii^{\prime }jj^{\prime }}\pi_{ij}\pi_{i^{\prime }j^{\prime }}{\left(c_{1}(X^{i},X^{i^{\prime }})-c_{2}(Y^{j},Y^{j^{\prime }})\right)}^{2} \end{array}$$

We define the *Manifold Gromov-Wasserstein Problem* to be the optimal coupling for (Alignment with Manifold Gromov-Wasserstein) given the Riemannian pull-back distances (6) $d^{M}(\cdot ,\cdot )$ and $d^{N}(\cdot ,\cdot )$ of each modality onto *E*.

Problem 2 (Manifold GW Problem.)

*Given pull-back distances (6)* $d^{M},d^{N}$*and distributions* $a\in \Delta_{n}$*over* ${\left(s^{i},X^{i}\right)}_{i=1}^{n}$*and* $b\in \Delta_{m}$*over* ${\left(t^{j},Z^{j}\right)}_{j=1}^{m}$*, Problem 2 is the optimization*

$$\pi^{⋆}\in \underset{\pi \in \Pi (a,b)}{\text{argmin}}\sum_{ii^{\prime }jj^{\prime }}{\left(d^{M}{\left(s^{i},s^{i^{\prime }}\right)}^{2}-d^{N}{\left(t^{j},t^{j^{\prime }}\right)}^{2}\right)}^{2}\pi_{ij}\pi_{i^{\prime }j^{\prime }}$$

Since $d^{M}$ and $d^{N}$ encode the spatial-feature product structure of $X^{i}=\varphi (s^{i})$ and $Z^{j}=\psi (t^{j})$ through the pullback metric, this offers a geometric solution to the Multimodal Alignment Problem 1.

In contrast to Fused Gromov-Wasserstein (FGW), MGW eliminates the need for a hyperparameter α in the objective that describes the trade off between spatial and feature similarity. FGW (Vayer et al. 2020) relies on a convex combination of Wasserstein ($C_{W}$) and Gromov-Wasserstein ($C_{GW}$) costs: ${\text{FGW}}_{\alpha }=\min_{\pi \in \Pi_{a,b}}\alpha \cdot C_{W}(\pi )+(1-\alpha )\cdot C_{GW}(\pi )$. This formulation forces a linear trade-off between dimensionally incompatible units (e.g. microns versus counts), making the problem definition dependent on a heuristic choice of α. Conversely, MGW poses a dimensionally consistent optimization without a hyperparameter in the objective. The learned Jacobians $J_{\varphi },J_{\psi }$ act as local operators converting spatial displacement into biological variation. A key advantage of the continuous formulation of MGW is that it defines a unique, intrinsic alignment problem. Consequently, while MGW uses a parameter *k* for graph construction and hyperparameters for the architecture of the neural field, these hyperparameters only govern the convergence of the learned neural fields and discrete geodesics to the true continuous signals (in Sobolev norm). Thus, these parameters are merely *approximation hyperparameters* and do not alter the underlying mathematical objective (Section 4, available as supplementary data at *Bioinformatics* online).

### 2.4 Theoretical properties and invariances

We highlight a few fundamental invariance and consistency properties of the Manifold Gromov-Wasserstein formulation.

- **(P1)** (Consistency with Spatial GW) If the neural fields φ and ψ are identities; i.e. φ(s)=s and ψ(t)=t, then $g^{M}(x)=g^{N}(x)=I$ and $d^{M}(x,x^{\prime })=d^{N}(x,x^{\prime })=\|x-x^{\prime }{\|}_{2}$. Thus, in the absence of information about the feature space, this offers an Occam’s Razor: (2) reduces exactly to the standard Gromov-Wasserstein problem on space *E*.
- **(P2)** (Spatial Isometry Invariance, Proposition 1) Problem 2 is invariant to translations $b\in R^{k}$, and orthogonal transformations $Q\in O_{k}=\{Q\in R^{k\times k}:Q^{⊤}Q=QQ^{⊤}=I_{k}\}$, so that solving (2) on $s^{i}$ is equivalent to solving on ${\tilde{s}}^{i}=Qs^{i}+b$.
- **(P3)** (Feature Isometry and Scaling Invariance, Proposition 2) Let $b,b^{\prime }\in M,N$ be any two constant vectors, let λ∈R:λ≠0 be a scaling, and $Q\in O_{d},U\in O_{p}$ any two global orthogonal feature transformations. Then, the solution to Problem 2 is invariant to transformations of the feature space of the form $\hat{\varphi }(x)=\lambda \cdot Q\varphi (x)+b$ and $\hat{\psi }(x)=\lambda \cdot U\psi (x)+b^{\prime }$.

MGW is invariant to the coordinate representation or parameterization of both the feature and spatial spaces, in contrast to coordinate-dependent formulations (Clifton et al. 2023, Tian et al. 2025). When trained on differing parametrizations of the feature and physical space, these guarantees hold and ensure MGW behaves robustly. Algorithm Theoretical Properties and Invariances summarizes MGW, with implementation details in Algorithm 1, available as supplementary data at *Bioinformatics* online and complexity described and profiled in Section 3, available as supplementary data at *Bioinformatics* online, Figs 5 and 6, available as supplementary data at *Bioinformatics* online.

**Algorithm 1** Manifold Gromov Wasserstein (MGW)

- *Learn neural fields.* Given samples $\left(s^{i},X^{i}\right)$ and $\left(t^{j},Z^{j}\right)$, learn maps $\varphi \equiv \varphi_{\theta }:E\to M$ and $\psi \equiv \psi_{\eta }:E\to N$:
  $$\underset{\theta }{\min}\frac{1}{2n}\sum_{i}\|\varphi_{\theta }(s^{i})-X^{i}{\|}_{2}^{2},\underset{\eta }{\min}\frac{1}{2m}\sum_{j}\|\psi_{\eta }(t^{j})-Z^{j}{\|}_{2}^{2}$$
- *Compute pull-back metrics.* For points in *E*:
  $$\begin{array}{cc} & g^{M}(s^{i})=J_{\varphi }{\left(s^{i}\right)}^{⊤}J_{\varphi }(s^{i}),g^{N}(t^{j})=J_{\psi }{\left(t^{j}\right)}^{⊤}J_{\psi }(t^{j}). \end{array}$$
- *Compute Riemannian geodesics.* Using $g^{M},g^{N}$, compute pairwise Riemannian distances $d^{M}(s^{i},s^{i^{\prime }})$ and $d^{N}(t^{j},t^{j^{\prime }})$ on *E*. Let $d_{ii^{\prime }}^{M}:=d^{M}{\left(s^{i},s^{i^{\prime }}\right)}^{2}$, resp. $d_{jj^{\prime }}^{N}$.
- *Solve Gromov–Wasserstein.*
$$\pi^{⋆}=\underset{\pi \in \Pi (a,b)}{\text{argmin}}\sum_{ii^{\prime }jj^{\prime }}{\left(d_{ii^{\prime }}^{M}-d_{jj^{\prime }}^{N}\right)}^{2}\pi_{ij}\pi_{i^{\prime }j^{\prime }}$$
- *Output coupling* $\pi^{⋆}.$

## 3 Results

We demonstrate the advantages of MGW on multiple alignment tasks, including Stereo-Seq spatiotemporal transcriptomics of mouse embryo Chen et al. (2022), Xenium and Visium spatial transcriptomics of colorectal cancer (Oliveira et al. 2025), and spatial metabolomics-transcriptomics from human striatum and kidney cancer (Hu et al. 2024, Vicari et al. 2024). For each experiment, we trained a pair of multi-layer perceptrons (MLPs) as our neural representations of each dataset (see Section 10.5 for details on the training and architecture). For the *k*-NN graph used to discretize the geodesics we set k=12. This reflects the local topology of the spatial grids (e.g. hexagonal arrays with six neighbors), ensuring robust connectivity beyond the first ring of neighborhoods while maintaining sparsity.

### 3.1 Spatiotemporal transcriptomics of mouse-embryo

As an initial baseline and ablation of MGW, we benchmark on unimodal transcriptomics-transcriptomics data from consecutive time-points E9.5–10.5, E10.5–11.5, E11.5–12.5, and E12.5–13.5 of the mouse embryo using the Stereo-Seq platform (Chen et al. 2022). To mimic a multi-modal alignment and to isolate the impact of the proposed MGW cost, we focus the evaluation against standard Gromov-Wasserstein (GW) baselines: GW on Euclidean spatial distances, GW on Euclidean feature distances, unbalanced GW on feature distances with moscot TranslationProblem (Klein et al. 2025), and unbalanced GW on raw (Euclidean) feature geodesic distances with SCOTv2 (Demetci et al. 2022a).

**Figure 3:**
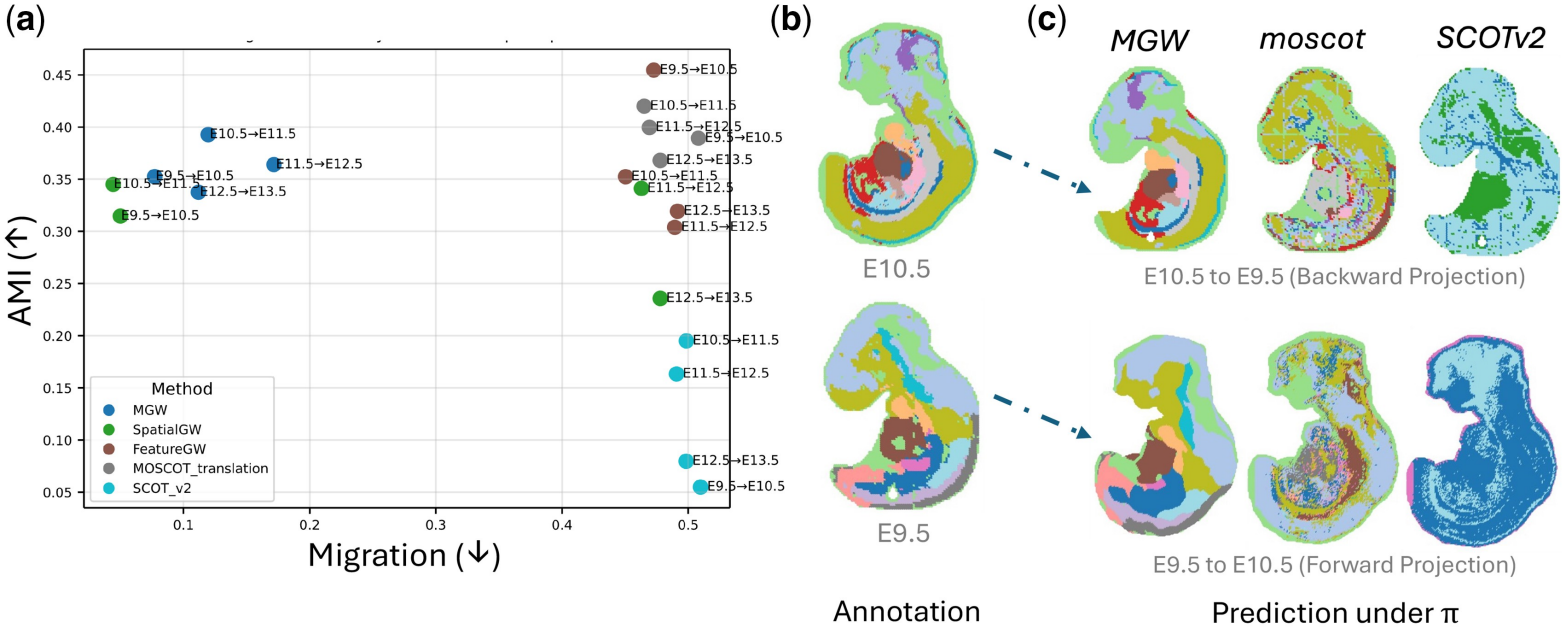
Comparison of Gromov-Wasserstein formulations on Stereo-Seq mouse-embryo timepairs E9.5–13.5 (Chen *et al*. 2022). (a) Comparison of alignments of pairs E9.5–10.5, E10.5–11.5, E11.5–12.5, and E12.5–13.5 in terms of migration distance and adjusted mutual information (AMI) with respect to published cell types. (b) Visualization of the annotated cell-types of Chen *et al*. (2022) and (c) the most-likely cell-type (50% confidence threshold) predicted in the forward time direction (E9.5→E10.5) and backward time direction (E10.5→E9.5) as transferred through each alignment π.

We assess alignments along two axes of quality: a metric of spatial realism, and a metric of feature alignment (Fig. 3a). For the spatial accuracy of the alignment, we compute the migration metric (Zeira et al. 2022, Liu et al. 2023, Halmos et al. 2025b) $\text{Mig}(\pi )=\sum_{i=1}^{n}\sum_{j=1}^{m}||s^{i}-t^{j}|{|}_{2}\pi_{ij}$: the average physical displacement required to align the tissues. For interpretability, we report the migration as percentage of the maximal extent of the slide. For the biological accuracy of the mapping, we compute the adjusted mutual information (AMI) of the predicted cell-types under the coupling against the ground-truth cell-type annotations (Chen et al. 2022).

We find that MGW achieves the best balance between spatial realism and feature alignment (Fig. 3a). MGW has expected cell-migration of 7.7%, 12.0%, 17.2%, and 11.2% of the slide compared to GW on spatial distances alone: 5.0%, 4.4%, 46.3%, and 47.8%. Moreover, MGW achieves higher AMI values than spatial GW for all pairs: 0.353, 0.393, 0.364, 0.338 versus 0.315, 0.345, 0.341, 0.236. Notably, for the latter time pairs (E11.5–12.5 and E12.5–13.5) MGW achieves even lower migration distances than spatial-only GW—in these cases, the non-convex spatial GW optimization can converge to a global flip relative to the ground-truth due to nearly spatially symmetric coordinates (Fig. 16, available as supplementary data at *Bioinformatics* online). By learning the metric for each modality, MGW can break such symmetries. Relative to performing GW on features alone, which yields unrealistically large migrations of 47.3%, 45.0%, 48.9%, and 49.2 % of the slide, MGW achieves much lower migration while maintaining comparable AMI values (0.353, 0.393, 0.364, 0.338 versus 0.455, 0.352, 0.304, 0.319). Likewise, compared to moscot, which produces migration distances of 50.8%, 46.5%, 46.9%, and 47.8% with AMI scores of 0.389, 0.420, 0.400, and 0.368, and to SCOTv2, which exhibits migrations of 51.0%, 49.9%, 49.1%, and 49.8% with markedly lower AMI (0.055, 0.195, 0.163, and 0.080), MGW attains minimal spatial displacement while maintaining high biological coherence, as shown in Fig. 3b and c which illustrates the predicted cell-types at ≥50% confidence. These results highlight the importance of spatial information to correctly align spatially structured tissues and underscore the value of aligning the intrinsic manifold as opposed to raw expression or spatial coordinates.

**Figure 4:**
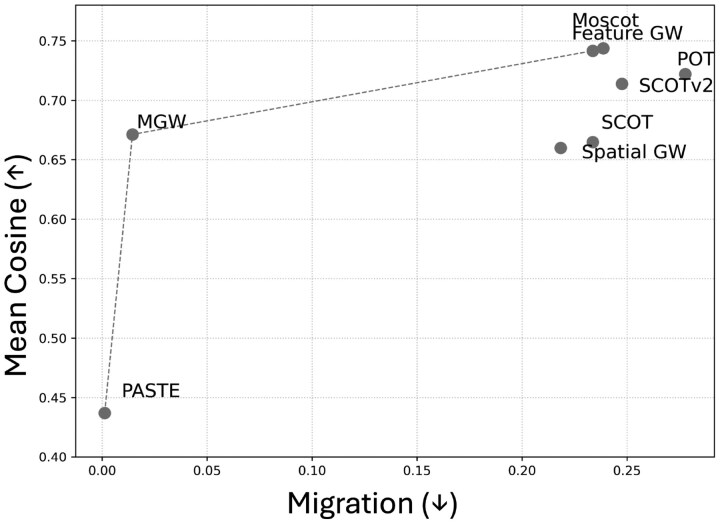
Cosine similarity and normalized migration for alignment of Visium and Xenium colorectal cancer data (Oliveira et al. 2025).

Lastly, a geometric ablation of MGW demonstrates that the neural pull-back outperforms its isolated components—unweighted spatial geodesics, neural field features, and discrete finite-difference approximations (Section 5.3, Fig. 4b, available as supplementary data at *Bioinformatics* online), confirming the necessity of the continuous Jacobian in capturing intrinsic tissue geometry.

### 3.2 Visium and Xenium alignment of colorectal cancer

We perform an alignment of two multi-modal sections of a 10x Genomics colorectal cancer (CRC) dataset from the same donor (Sample P2 CRC) (Oliveira et al. 2025). This dataset includes a Visium CytAssist v2 section and a Xenium spatial transcriptomics section profiling the same tissue. While both datasets measure gene expression, they represent distinct measurement types: in Visium one performs sequencing of the full transcriptome (18k genes), while Xenium performs FISH imaging on a smaller panel of 422 genes. We benchmark 8 techniques on this dataset: MGW, Spatial GW, Feature GW, moscot (Klein et al. 2025), SCOT (Demetci et al. 2022b), SCOTv2 (Demetci et al. 2022a), PASTE2(FGW) (Liu et al. 2023), and POT (Flamary et al. 2021). We measure alignment quality via (i) mean expression cosine similarity between common Xenium-Visium genes, and (ii) the spatial migration distance between aligned spots. Strong alignments maintain high cosine similarity in aligned cells, while also minimizing spatial distortion and crossings in the alignment.

We find that MGW achieves the best balance with high cosine similarity (mean 0.671 and median 0.689) and low migration distance (average 1.4% of the slide) (Fig. 4, Table 3, available as supplementary data at *Bioinformatics* online). Most of the other techniques—including Feature GW, moscot, SCOT, and SCOTv2– align based on feature-information alone and ignore the constraint of spatial context. As a result, they exhibit higher mean cosine scores 0.744/0.742/0.665/0.714, respectively, but also a highly unrealistic amount of spatial migration of 23.9 %/23.4%/23.4%/24.8%, respectively, of the spatial extent of the slide for the cell-cell mapping (Fig. 4, Fig. 9, available as supplementary data at *Bioinformatics* online). As PASTE2 is not a multi-modal alignment method, it only uses spatial information and achieves a more realistic quantity of migration of only 0.1% of the slide but also a low expression cosine score of 0.437 indicating poor alignment of the transcription. Thus, MGW is the only technique with cosine-similarity at the level of feature-only methods and spatial distortion at the level of spatial-only methods.

### 3.3 Metabolomics and transcriptomics alignment of human clear cell renal cell carcinoma (ccRCC)

We evaluated MGW on two tissue slices from clear-cell renal cell carcinoma (ccRCC), one assayed with a AFADESI-MSI spatial metabolomics and the other with 10x Genomics Visium spatial transcriptomics [dataset Y7_T from (Hu et al. 2024)]. We benchmark on the task introduced in (Tian et al. 2025), which consists of: (i) aligning the multi-modal spatial metabolomic-transcriptomic datasets, (ii) projecting the metabolomic profiles onto the transcriptomic coordinates through the alignment, and (iii) computing joint-embeddings with the variational autoencoder (VAE) architecture of SpatialMeta (Tian et al. 2025). We compare MGW to multi-modal single-cell optimal-transport (OT) based techniques SCOT (Demetci et al. 2022b), SCOTv2 (Demetci et al. 2022a), and moscot Translation (Klein et al. 2025), and to the non-OT based spatial metabolomics-transcriptomics method SpatialMeta (Tian et al. 2025), which introduced an Alignment Module based on STAlign (Clifton et al. 2023) applied to the spatial coordinates. We also include Spatial-only GW and Feature-only GW as baselines. We ensure the training and architecture of the VAE are identical for all methods and repeat the entire evaluation pipeline, including alignment and training, across random seeds to ensure consistency. A complete summary of all metrics is provided in Table 1, available as supplementary data at *Bioinformatics* online, and further details on our evaluation procedure and implementation may be consulted in Section 5, available as supplementary data at *Bioinformatics* online.

**Figure 5:**
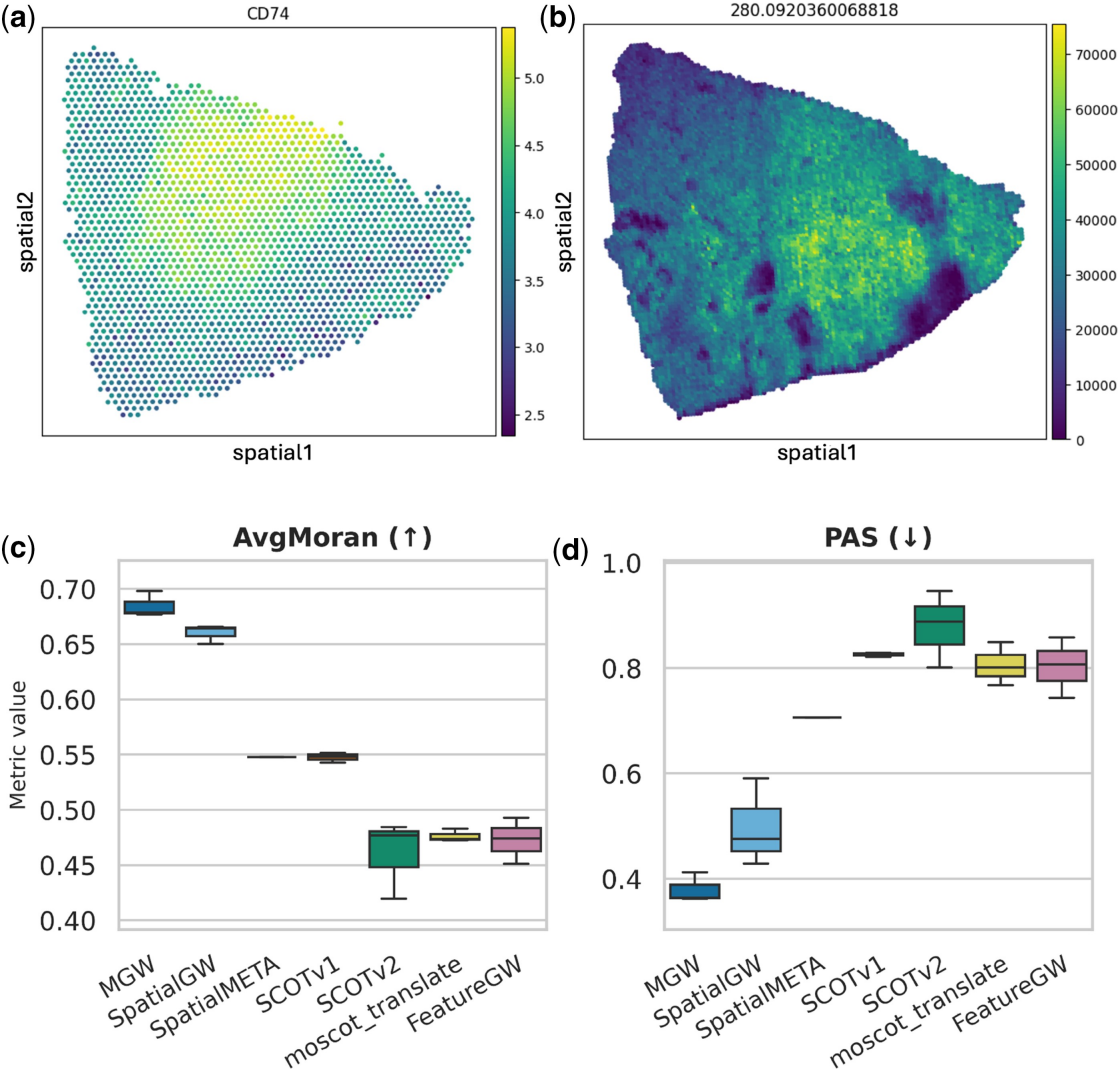
Cross-modal spatial transcriptomics and metabolomics alignment evaluation of MGW, spatial transcriptomics-metabolomics method SpatialMeta, and other OT baselines on the Y7_T slice from the ccRCC dataset (Hu *et al*. 2024). (a) The spatial distribution of gene *CD74* and (b) an associated metabolite with high Moran’s I. (c) Average Moran’s I on the two modalities and (d) PAS reported across three random seeds.

We find MGW demonstrated the highest average spatial coherence across all methods, as quantified by Moran’s I across both modalities (0.6845 ± 0.0134) (Fig. 5c). Moran’s I assesses whether the top spatial transcriptomics and spatial metabolomics marker features selected from the Leiden clusters exhibit spatially smooth, region-specific expression patterns. MGW substantially outperforms SpatialMeta (0.5478), which is specifically designed for spatial transcriptomic and metabolomic alignment.

MGW also outperforms the other methods on all three spatial continuity metrics used in (Tian et al. 2025) (Table 1, available as supplementary data at *Bioinformatics* online). For example on PAS score, which quantifies how frequently a spot has neighbors belonging to a different cluster (see Section 5, available as supplementary data at *Bioinformatics* online), MGW achieved the lowest (best) value (Fig. 5d), averaging 0.3797 across seeds, exceeding SpatialMeta (0.7063) as well as SCOT, SCOTv2 and moscot with scores of 0.8255, 0.8784, and 0.8058. In addition, the scores exceed those of Feature and Spatial GW (0.8029 and 0.4985, respectively). Techniques which aligned using spatial coordinates alone, such as SpatialMeta (which uses STAlign) and Spatial Gromov-Wasserstein, exhibited stronger performance on this task than ones relying on feature-information alone. This reflects the similarity of the aligned spatial grids (Fig. 5a and b).

### 3.4 Metabolomics-transcriptomics alignment of striatum

**Figure 6:**
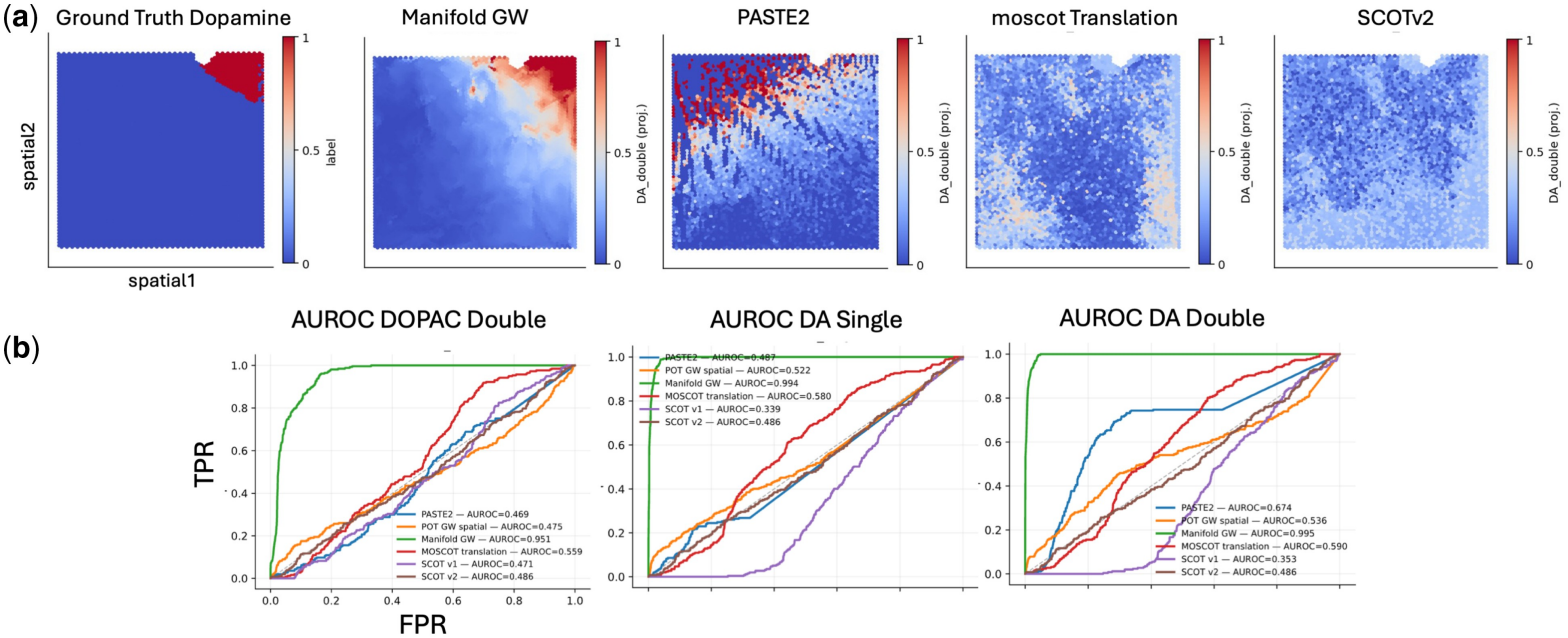
Evaluation of Dopamine recovery in the Striatum. (a) (Left) Published (“Ground-truth”) dopamine labels on Visium slide (Vicari et al. 2024). (Right) Normalized barycentric projection of doubly derivatized dopamine onto Visium slide across methods. (b) AUROC curves for the overlap between the locations of Dopamine metabolites DA Single (singly derivatized m/z=421.19), DA double (doubly derivatized m/z=674.28), 3MT (dopamine-breakdown product 3-Methoxytyramine, m/z=435.21), and DOPAC double (dopamine-breakdown product m/z=698.24) and the annotated locations of dopamine-associated neurons on Visium spatial transcriptomics slide for each method.

Lastly, we align two post-mortem sections from the striatum region of the human brain. One section contains spatial profiling of low molecular weight metabolites determined using (MALDI)-MSI mass-spectrometry, and the other section contains spatial profiling of mRNA transcripts measured using the 10x Genomics Visium technology (Vicari et al. 2024). The sections are derived from a donor with Parkinson’s disease, a neurodegenerative disorder characterized by dopamine depletion in the striatum. To evaluate MGW in comparison to other techniques, we assess the correspondence between the concentration of dopamine and its immediate breakdown products at various mass: charge ratios m/z in the spatial metabolomics dataset compared to the published annotations of Visium spots by dopamine-positive identity provided in Vicari et al. (2024) (Fig. 6a). Specifically, Vicari et al. (2024) identifies 4 key dopamine metabolites, including DA Single (singly derivatized dopamine) at m/z=421.19, DA double (doubly derivatized dopamine) at m/z=674.28, the dopamine-breakdown product 3MT (3-Methoxytyramine) at m/z=435.21, and the dopamine-breakdown product DOPAC double at m/z=698.24. Dopamine-associated neurons were defined using the dopamine-high (Cd) annotations of the spatial transcriptomics slide.

We compared MGW to multi-modal single-cell alignment techniques moscot Translation (Klein et al. 2025), SCOT (Demetci et al. 2022b), and SCOTv2 (Demetci et al. 2022a), as well as two spatial-only baselines: PASTE2(FGW) with spatial information alone (Liu et al. 2023), and spatial-only GW. We choose the latter baselines in order to assess that the efficacy of the MGW alignment is not merely due to spatial information. Each method produces a coupling π between the locations on the metabolomics and spatial transcriptomics slices. We compute a barycentric projection (Peyré and Cuturi 2019) of the metabolite intensities through each π onto the ST coordinates. All methods except PASTE2, which performs partial alignment, transfer the full dopamine mass-intensity to the ST slide. We quantify alignment accuracy as the overlap between projected dopamine intensities from the metabolomics slices and the annotated binary dopamine-high spot labels on the transcriptomics slice, and computed the AUROC and AUPRC as a function of the predicted metabolite scores (Fig. 6b). As the dopamine metabolites and dopamine-high spots form a weak, sharply localized signal (see Fig. 8, available as supplementary data at *Bioinformatics* online for unscaled, raw-intensities), this benchmark assesses high-precision mapping of this small but biologically significant region. Low scores indicate failure to accurately map this local signal rather than poor global alignment.

MGW achieves the highest scores across all four metabolites (Table 4, available as supplementary data at *Bioinformatics* online, Fig. 6b). Specifically, the AUROC and AUPRC scores for 3MT, DA double, DA single, and DOPAC double were 0.991/0.846, 0.995/0.907, 0.994/0.882, and 0.951/0.455, respectively. These consistently exceeded the scores of other methods by large margins—e.g. moscot Translation achieved 0.615/0.063, 0.590/0.058, 0.580/0.057, and 0.559/0.054, while the strongest spatial baseline PASTE2 reached 0.667/0.084, 0.674/0.090, 0.487/0.055, and 0.469/0.046. The alignment of Spatial GW performed near chance (AUROC ∼0.52). Visualizations of the projections (Fig. 6a) corroborate the observed quantitative trend: MGW correctly projects the dopamine metabolites onto the ground-truth dopamine-annotated regions with high spatial localization and accuracy. Other methods yield diffuse or incorrectly localized patterns, indicating that accurate spatial multiomic alignment requires both spatial and feature modalities. In this high-precision, localized prediction task, MGW is the only method which projects dopamine metabolites to the dopamine-annotated neurons.

## 4 Discussion

We introduce Manifold Gromov-Wasserstein (MGW) to align spatial multiomics by learning modality-specific Riemannian metrics via neural fields. This approach compares intrinsic structure on a common spatial base, avoiding any hyperparameters that balance dimensionally inconsistent costs. We demonstrate the generality of MGW across diverse sequencing (Visium, Stereo-seq), imaging (Xenium), and mass-spectrometry metabolomics (MALDI-MSI, AFADESI-MSI) modalities.

MGW provides a geometrically principled distance that leverages the product structure M×E (respectively N×E) of spatial omics data, and naturally integrates both physical and feature domains. In contrast, existing techniques are limited by their approach to this integration. One class of methods rely solely on modality features M,N (e.g. moscot translation, SCOT, SCOTv2) and discard spatial context needed for tissue architecture. Another class of methods rely primarily on spatial information *E* (SpatialMeta, STAlign) and yield spatially coherent alignments that miss molecular correspondences. Finally, a third class of methods (e.g. PASTE, and PASTE2) fuse combinations of spatial and feature costs with a heuristic trade-off between spatial and feature similarity but lack a well-defined cross-modality distance for different features spaces M≠N.

There are several limitations of MGW which present opportunities for future work. First, alignment quality is constrained by the fidelity of the learned Riemannian metric and thus the underlying neural fields. While we used MLPs, exploring architectures which better approximate the true biological signals φ,ψ is a natural future direction. Second, unshared modality-specific features may confound metric estimation. While we use CCA pre-processing (Section 5.8, available as supplementary data at *Bioinformatics* online), explicitly modeling shared subspaces could improve robustness. Third, while we used balanced optimal transport in our experiments to highlight the effect of the learned Manifold-GW cost alone, MGW supports unbalanced OT via ott-jax Cuturi *et al*. (2022), and optimal transport variants such as unbalanced (Schiebinger et al. 2019, Demetci et al. 2022a, Klein et al. 2025), semi-relaxed (Halmos et al. 2025b), and partial (Liu et al. 2023) OT often improve alignment robustness.

Future evaluations could extend MGW to other modalities such as histology (H&E) or weakly structured spatial tissue distributions where geometric signals may be less pronounced. Additionally, the learned Riemannian distances may prove advantageous for highly non-linear transformations such as spatiotemporal transcriptomics. In addition, our ablations suggest that the MGW Riemannian distances constitute a stronger GW term than either spatial or feature distances alone (Section Spatiotemporal Transcriptomics of Mouse-Embryo), suggesting FGW frameworks may benefit from adopting the MGW cost.

Manifold Gromov-Wasserstein demonstrates that combining optimal transport with costs learned through deep Riemannian metric-learning provides a powerful and general approach for aligning heterogeneous spatial-omics datasets. More broadly, our results underscore that learning intrinsic geometry provides a powerful unifying principle for aligning nonlinear manifold data across the spatial multi-omic universe.

## Author contributions

Peter Halmos (Conceptualization, Methodology, Software, Investigation, Writing—original draft, Writing—review and editing), Yufan Xia (Software, Investigation, Writing—review and editing), and Benjamin J. Raphael (Investigation, Writing—review and editing, Supervision)

## Supplementary Material

btag220_Supplementary_Data

## Data Availability

The spatial multiomics datasets analyzed in this study are publicly available from their original publications and associated data repositories: - The spatiotemporal mouse transcriptomics Stereo-Seq data (Chen 2022) is available via the MOSTA database. - The Visium and Xenium colorectal cancer (CRC) dataset (Oliveira 2024) is available from the 10x Genomics spatial genomics data repository. - The MALDI-MSI metabolomics and Visium transcriptomics dataset of human striatum (Vicari 2023) is available at Mendeley Data under accession: https://data.mendeley.com/datasets/w7nw4km7xd/1 - The AFADESI-MSI metabolomics and Visium transcriptomics dataset of human clear cell renal carcinoma (ccRCC) (Tian 2025) is available at Zenodo under accession (https://zenodo.org/records/14986870). The open-source Python implementation of MGW, along with tutorials and scripts to reproduce the experiments described in this manuscript, is freely available on GitHub at https://github.com/raphael-group/MGW and archived on Zenodo at DOI: 10.5281/zenodo.20074368.
