# Supplementary material for: Riemannian metric learning for alignment of spatial multiomics
Source: Bioinformatics. 2026 Jul 7;42(Suppl 1):btag220. doi: 10.1093/bioinformatics/btag220 (PMC13340267; doi:10.1093/bioinformatics/btag220)
Supplement: btag220_Supplementary_Data [file btag220_supplementary_data.pdf]

Appendix

Pull-back Metrics and Base-Space Parametrization

Suppose we are given two Riemannian manifolds $(\mathcal{M}, g)$ and $(\mathcal{N}, h)$ and measures (datasets) $\mu \in \mathcal{P}(\mathcal{M})$, $\nu \in \mathcal{P}(\mathcal{N})$ supported on each space: for instance, for single-cell transcriptomics $\mathcal{M}$ could represent an embedded sub-manifold of the d-dimensional measurements of transcript features $\mathcal{M} \subset \mathbb{R}^d$, and for a distinct modality, e.g. metabolomics, $\mathcal{N}$ could represent a sub-manifold of the p-dimensional measurements of metabolites $\mathcal{N} \subset \mathbb{R}^p$ where we may have that $p \neq d$ and $\dim(\mathcal{M}) \neq \dim(\mathcal{N})$. In general, it may appear to be a formidable task to align such datasets across such incomparable spaces, with no "natural" notion of distance immediately evident.

However, suppose that we instead take measurements on the product space $\mathcal{M} \times E$ and $\mathcal{N} \times E$ for some common base manifold (E, g^E). This may correspond to Euclidean space $E = \mathbb{R}^2$ or $E = \mathbb{R}^3$, e.g. when one reads the product of feature vectors with spatial coordinates as in spatial transcriptomics or spatial metabolomics. Recently, a remarkable line of work Chitra et al. (2025) have shown that one may learn an implicit neural representation of such product datasets. In particular, Chitra et al. (2025) shows that one may represent data $(E, \mathcal{M})$ as a smooth and differentiable map $\varphi \in C^k$ from a base-space E into a feature space $\mathcal{M}$, $\varphi : E \to \mathcal{M}$. In other words, given spatial coordinates s^i and product vector $p^i = (s^i, X^i) \in E \times \mathcal{M}$ one may instead represent $X^i := \varphi(s^i)$ and thus $p^i = (\mathrm{id}, \varphi) \circ s^i$ for smooth, differentiable φ: a spatial transcriptomics dataset may be implicitly represented as a smooth function of its spatial coordinates. Thus, we state a first key assumption.

Assumption 1 Given $(\mathcal{M}, g)$ and $(\mathcal{N}, h)$ Riemannian, a common base manifold (E, g^E), and points $p^i = (s^i, X^i) \in E \times \mathcal{M}$ and $q^j = (s^j, Z^j) \in E \times \mathcal{N}$ we assume there there exist two C^k local-parametrizations

$$\varphi : E \to \mathcal{M}, \quad \psi : E \to \mathcal{N} \tag{8}$$

which satisfy

$$X^i = p^i \mid_{\mathcal{M}} = \varphi(p^i \mid_E) = \varphi(s^i), \quad Z^j = q^j \mid_{\mathcal{N}} = \psi(q^j \mid_E) = \psi(s^j).$$

As in Chitra et al. (2025), we optimize for such a pair of differentiable mappings with a neural network encoding each dataset implicitly. One solves this pair of maps φ, ψ as

$$\varphi := \varphi_\theta, \quad \theta = \arg\min_\theta \frac{1}{2n} \sum_{i=1}^{n} \left\| \varphi_\theta(s^i) - X^i \right\|_2^2,$$
$$\psi := \psi_\eta, \quad \eta = \arg\min_\eta \frac{1}{2m} \sum_{j=1}^{m} \left\| \psi_\eta(s^j) - Z^j \right\|_2^2$$

Given explicit maps φ, ψ from the base E into $\mathcal{M}$ and $\mathcal{N}$ with associated metrics on each space, there are natural vector fields defined through the mappings and a natural metric. In particular, for a vector field on E, $V \in \mathfrak{X}(E)$, one has associated push-forward vector fields on $\mathcal{M}, \mathcal{N}$ given by

$$\varphi_* V(f) := V(f \circ \varphi), \quad \psi_* V(g) := V(g \circ \psi) \tag{9}$$

defined by $\langle df, \varphi_* V \rangle_{T_{\varphi(p)}\mathcal{M}} = \langle d(f \circ \varphi), V \rangle_{T_p E}$ for any test function $f \in C^\infty(\mathcal{M})$ (resp. $\mathcal{N}$). With a correspondence between spaces, we may measure manifold distances on each space with a *pull-back metric.*

Definition 3 (Pull-Back Metric.) Given manifolds (E, g^E), $(\mathcal{N}, h)$ and mapping $\psi : E \to \mathcal{N}$, the pull-back metric of h is the symmetric $(2, 0)$-tensor on E defined by

$$(\psi^* h)(X, Y) = h(\psi_*(X), \psi_*(Y)) \tag{10}$$

From the duality of $\langle df, \psi_* V \rangle_{T_{\psi(p)}\mathcal{M}} = \langle d(f \circ \psi), V \rangle_{T_p E}$, one identifies

$$h(\psi_* V, \psi_* V) = g^E(V, (d\psi^\top \cdot h(\psi(p)) \cdot d\psi) \circ V)$$

so that one may write this in terms of the Jacobian as

$$(\psi^* h)_{\alpha,\beta}(p) = h_{ij}(\psi(p)) \frac{\partial \psi^i}{\partial x^\alpha} \frac{\partial \psi^j}{\partial x^\beta},$$
$$x_\alpha x_\beta \, (\psi^* h)_{\alpha,\beta}(p) = \|x\|^2_{g_p^M},$$

For metric $g_p^M = D\psi(p)^\top \, h(\psi(p)) \, D\psi(p) : T_p E^* \times T_p E \to \mathbb{R}_+$ defined at any point $p \in E$.

Now, suppose for simplicity that g and h are taken to be the standard Euclidean metrics $g_{\alpha\beta} = \delta_{\alpha\beta}$ in their respective spaces (i.e. in normal coordinates). Then, given a non-trivial pair of mappings φ, ψ one can find the following pull-back of both $\mathcal{M}, \mathcal{N}$ to the common Euclidean space E:

$$\underbrace{g^M(p) = D\varphi(p)^\top D\varphi(p)}_{\text{Manifold M Pull-Back}}, \quad \underbrace{g^N(q) = D\psi(q)^\top D\psi(q)}_{\text{Manifold N Pull-Back}}$$

Thus, we arrive at a remarkable identification: if we measure in a product space $E \times \mathcal{M}$ and $E \times \mathcal{N}$, and the pull-back maps (φ, ψ) exist, one may pull manifold-distances back to a *common* base space. Observe that g^M and g^N are *tensor fields* over E: each coordinate p, q has its own metric tensor $g^M(p)$ and $g^N(q)$, which may vary signficantly over E.

Metric Distances and Energy

Let us recall the fundamental notion of distance used with respect to a metric g. The infinitesimal unit ds is known as the line element and gives an infinitesimal unit of arc length (distance). To extend this to distances over curves, define any curve over the manifold by $\gamma : [0, 1] \to E$, and the line-element of this curve at any time t and point $\gamma(t)$ by

$$ds = \sqrt{g_{ij}(\gamma(t))(\dot{\gamma}, \dot{\gamma})} dt$$

one measures the distance of a curve γ over time t with the arc-length integral

$$\int_\gamma ds = \int_0^1 \sqrt{g_{ij}(\gamma(t))(\dot{\gamma}, \dot{\gamma})} dt = L_g(\gamma)$$

For local coordinates $X = (x^1, \cdots, x^n)$ one defines the infinitesimal squared-distance in g by

$$ds^2 = g_{ij}(X) \, dx^i dx^j,$$

So that the extension for a curve γ is $ds^2(\gamma) = g_{ij}(\gamma(t)) \, \dot{\gamma}^i \dot{\gamma}^j$. The *energy* of a curve γ is defined as the integral of this squared-distance unit along the curve γ

$$E_g(\gamma) = \frac{1}{2} \int_\gamma ds^2 = \frac{1}{2} \int_0^1 g(\gamma)(\dot{\gamma}, \dot{\gamma}) \, dt := \frac{1}{2} \int_0^1 \|\dot{\gamma}\|^2_{g(\gamma)} dt$$

Suppose one normalizes γ to be parametrized by arc-length, i.e. the unique constant speed parametrization of γ so that $\|\dot{\gamma}\|_2 = 1$, then

one may define a geodesic distance between any points x^a, x^b by the minimal distance between these points over all curves γ connecting them:

$$d_g(x^a, x^b) = \inf_{\substack{\gamma:[0,1]\to E,\\ \gamma(0)=x^a,\gamma(1)=x^b}} \left\{ L_g(\gamma) = \int_0^1 \sqrt{g(\gamma(t))(\dot{\gamma},\dot{\gamma})}dt \right\}$$

Which, for constant speed curves, is related to the energy by

$$d_g(x^a, x^b) = \inf_{\gamma\,:\,x^a\to x^b} L_g(\gamma) = \inf_{\gamma\,:\,x^a\to x^b} \sqrt{2E_g(\gamma)}.$$

The argmin of these problems is the unique constant-speed γ^* from x^a to x^b, and the minimum value of $d_g(x^a, x^b)$ is referred to as either a Riemannian distance or a geodesic distance between the pair of points. Thus, given a potentially point-dependent metric g on $\mathcal{M}$, one may define a geodesic distance between any pair of points. In the standard Euclidean case, the definitions above automatically recover the standard Euclidean distance and distance-squared, where $g_{ab}(x) = g_{ab} := \delta_{ab}$ reduces to a trivial constant metric which lacks any dependence on the location of x on $\mathcal{M}$.

Manifold Alignment via Geodesic Pull-Back Distances

Using the notions of distance above, we provide a formulation for the Gromov-Wasserstein problem (Definition 5) which offers a geometric unification of the alignment problem between spaces using the pull-back. In particular, this cost relies on geodesic pull-back distances under the pull-back metrics g^M, g^N over the common base E.

Definition 4 (Riemannian Pull-Back Distances.) Suppose we are given two feature manifolds $(\mathcal{M}, g)$, $(\mathcal{N}, h)$, a common base manifold (E, g^E), and points $p^i = (s^i, X^i) \in E \times \mathcal{M}$ and $q^j = (s^j, Z^j) \in E \times \mathcal{N}$. Further, suppose that Assumption 1 holds with associated maps $\varphi : E \to \mathcal{M}$, $\psi : E \to \mathcal{N}$. We define the M-distance and N-distance of any two points $x^s, x^t \in E$ to be the associated geodesic pull-back distances:

$$d^M(x^s, x^t) = \inf_{\substack{\gamma:[0,1]\to E,\\ \gamma(0)=x^s,\ \gamma(1)=x^t}} \left\{ \int_0^1 \sqrt{g^M(\gamma(t))(\dot{\gamma},\dot{\gamma})}dt \right\},$$
$$d^N(x^s, x^t) \quad \text{resp.}$$

For pull-back metrics g^M, g^N defined by

$$g^M(p) = D\varphi(p)^\top D\varphi(p), \quad g^N(q) = D\psi(q)^\top D\psi(q)$$

Now, given appropriate notions of pairwise distance in the common Euclidean space E, we may define the associated Gromov-Wasserstein (GW) problem Mémoli (2011) as the natural notion of metric distortion between two spaces with two metrics.

Definition 5 (Manifold (Pull-back) Gromov-Wasserstein.) Given Riemannian pull-back distances $d^M(\cdot,\cdot)$ and $d^N(\cdot,\cdot)$ (Definition 4), define the associated squared distortions to be $C^M(\cdot,\cdot) := d^M(\cdot,\cdot)^2$, and $C^N(\cdot,\cdot) := d^N(\cdot,\cdot)^2$. For two distributions $\mu^M \in \mathcal{P}(E)$ and $\mu^N \in \mathcal{P}(E)$, the manifold pull-back GW Distance between μ^M and μ^N, is

$$\mathrm{GW}_E^{(M,N)}(\mu^M, \mu^N) \tag{11}$$
$$= \min_{\pi\in\Pi(\mu^M,\mu^N)} \mathbb{E}_{(x,z),(x',z')\sim\pi\otimes\pi}[C^M(x,x') - C^N(z,z')]^2 \tag{12}$$

In other words, this is the standard Gromov-Wasserstein distance with Riemannian distance matrices built from Definition 4.

The corresponding discrete problem for Definition 4 is given for two datasets of points $(s^i)_{i=1}^n \subset E$ and $(t^j)_{j=1}^m \subset E$ in the Euclidean base-space by

$$\min_{\mathbf{P}\in\Pi(\boldsymbol{a},\boldsymbol{b})} \sum_{ii'}\sum_{jj'} (C^M(s^i, s^{i'}) - C^N(t^j, s^{j'}))^2\ \mathbf{P}_{ij}\mathbf{P}_{i'j'}$$
$$:= \min_{\mathbf{P}\in\Pi(\boldsymbol{a},\boldsymbol{b})} \sum_{ii'}\sum_{jj'} (C^M_{ii'} - C^N_{jj''})^2\ \mathbf{P}_{ij}\mathbf{P}_{i'j'}$$

We summarize this discrete formulation in Problem 2. In the next section, we discuss a number of desirable properties and invariances of Definition 5 which makes it a natural option for aligning data that admits a product structure across two spaces.

Properties of the Gromov-Wasserstein Distance on the Metric Pull-back.

Consistency under identity mapping

Observe that if $\varphi, \psi :=$ id so that $\varphi(s), \psi(t) := s, t$ – i.e., there is no feature modality, one has that

$$g^M(p) = D\varphi(p)^\top D\varphi(p) := \mathbb{1}^\top\mathbb{1} = \mathbb{1},$$
$$g^N(q) = D\psi(q)^\top D\psi(q) = \mathbb{1}^\top\mathbb{1} = \mathbb{1}$$

so that the metric tensor is the identity, and thus the geodesic distances become Euclidean distances, $d^M(x^s, x^t) = \|x^s - x^t\|_2$. As a consequence, (11) simply reduces to a Gromov-Wasserstein problem on the raw spatial distances. Thus, (11) is consistent: in the case that no feature modality is present it recovers standard Gromov-Wasserstein on the spatial distances alone.

Spatial Invariances

Proposition 1 (Spatial Invariances of Manifold Gromov-Wasserstein.) *Problem 2 is invariant to arbitrary translations $b \in \mathbb{R}^k$, and orthogonal transformations $Q \in \mathcal{O}_k = \{Q \in \mathbb{R}^{k\times k} : Q^\top Q = QQ^\top = \mathbf{I}_k\}$, so that solving 2 on s^i is equivalent to solving on $\tilde{s}^i = Qs^i + b$.*

Proof Let $g^N = D\varphi^\top D\varphi$ on a Euclidean manifold $E = \mathbb{R}^k$. To assess spatial invariances, we consider which maps $\Phi : E \to E$ applied to the points $x \to \Phi(x)$ maintain the solution of the objective. Let us consider the action of the class of rigid-body transformations for arbitrary translations $b \in \mathbb{R}^k$, and orthogonal transformation $Q \in \mathbb{R}^{k\times k} : Q^\top Q = QQ^\top = \mathbf{I}_k$. Suppose one learns the maps $\hat{\varphi}, \hat{\psi}$ over a rigid-body transformation of the coordinates $y = Qx + b$, $x = Q^\top(y - b)$ and denote the maps over the original coordinates by φ, ψ. Since the features are identical under rotation of the spatial grid, one has that within the compact set $\Omega \subset E$ our points are supported on:

$$\hat{\varphi}(Qx + b) = \varphi(x), \quad \hat{\psi}(Qx + b) = \psi(x)$$

and thus for the modified metric tensor $\hat{g}^M$ one identifies

$$D\hat{\varphi}(y) \circ Q = D\varphi(x), \quad D\hat{\varphi}(y) = D\varphi(x)\,Q^\top$$
$$\hat{g}^M(y) = D\hat{\varphi}(y)^\top D\hat{\varphi}(y)$$
$$= Q\,D\varphi(x)^\top D\varphi(x)\,Q^\top = Q\,g^M(x)\,Q^\top$$

implying that for any curve γ and its rigid transformation $\gamma_* = Q\gamma + b$ one has $\dot{\gamma}_* = Q\dot{\gamma}$ so that

$$\begin{aligned}\hat{g}^M(y)(\dot{\gamma}_*, \dot{\gamma}_*) &= \hat{g}^M(y)(Q\dot{\gamma}, Q\dot{\gamma})\\ &= (Q\dot{\gamma})^\top Q g^M(x) Q^\top (Q\dot{\gamma}) = \dot{\gamma}^\top Q^\top Q g^M(x) Q^\top Q \dot{\gamma}\\ &= \dot{\gamma}^\top g^M(x)\dot{\gamma} = g^M(x)(\dot{\gamma}, \dot{\gamma})\end{aligned}$$

So that infinitesimal line elements coincide, and

$$ds^2_{\hat{g}^M}(\gamma_*) = ds^2_{g^M}(\gamma)$$

and integrating along curves yields that the geodesic distance in M (resp. N) is invariant under rigid motions

$$d^M_{\hat{g}}(Qu_1 + b, Qu_2 + b) = d^M_g(u_1, u_2)$$

□

Feature Invariances

Similarly to the spatial case, for the feature case we evaluate which transformations T_φ, T_ψ of the feature maps φ, ψ maintain the modified objective.

Proposition 2 (Feature Invariances of Manifold Gromov-Wasserstein.) *Let $b, b' \in \mathcal{M}, \mathcal{N}$ be any two constant vectors, let $\lambda \in \mathbb{R} : \lambda \neq 0$ be a scaling, and $Q \in \mathcal{O}_d, U \in \mathcal{O}_p$ any two global orthogonal feature transformations. Then, the solution to Problem 2 is invariant to transformations of the feature space of the form*

$$\hat{\varphi}(x) = \lambda \cdot Q\varphi(x) + b, \quad \hat{\psi}(x) = \lambda \cdot U\psi(x) + b'$$

Proof Let us focus on the following class of transformations for any constant vector $b, b' \in \mathcal{M}, \mathcal{N}$, a scaling $\lambda \in \mathbb{R} : \lambda \neq 0$, and a global orthogonal feature transformation $Q \in \mathcal{O}_d, U \in \mathcal{O}_p$

$$\hat{\varphi}(x) = \lambda \cdot Q\varphi(x) + b, \quad \hat{\psi}(x) = \lambda \cdot U\psi(x) + b'$$

One may verify that the Jacobian is necessarily translation-invariant, yielding

$$\begin{aligned}D\hat{\varphi}(x) = \lambda Q D\varphi(x), \quad \hat{g}^M(x) &= (\lambda Q D\varphi(x))^\top (\lambda Q D\varphi(x))\\ = \lambda^2 D\varphi(x)^\top Q^\top Q D\varphi(x) &= \lambda^2 D\varphi(x)^\top D\varphi(x) = \lambda^2 g^M(x)\end{aligned}$$

With infinitesimal line-elements differing by a homogeneous constant, and implying

$$d^M_{\hat{g}}(u_1, u_2) = \lambda \cdot d^M_g(u_1, u_2)$$

So that, applying the same reasoning to $\hat{\psi}$, we have

$$\begin{aligned}&(\lambda^2 \cdot d^M_g(u_1, u_2)^2 - \lambda^2 \cdot d^N_g(u_1', u_2')^2)^2\\ &= \lambda^4 (d^M_g(u_1, u_2)^2 - d^N_g(u_1', u_2')^2)^2\end{aligned}$$

And have $\mathrm{GW}_{\hat{\varphi}, \hat{\psi}} = \lambda^4 \cdot \mathrm{GW}_{\varphi, \psi}$, so that the solution is unchanged to this class of transformation and is conformally invariant to the change of metric $g \to \lambda^2 g$. □

Computational Complexity

The complexity of `MGW` depends on its three algorithmic components: (1) estimation of the neural fields φ, ψ, (2) computation of Riemannian geodesics, and (3) Gromov-Wasserstein optimal transport alignment of the geodesics. For (1), we perform mini-batch training of a multi-layer perceptron in `torch`. Viewing the dimension d as constant, this scales linearly in the sample complexity as $\tilde{O}(nd)$. For (2) while all pairs shortest paths (APSP) scales cubically in general with algorithms such as Floyd-Warshall, we employ a sparse k-NN graph for which Dijkstra's exhibits $\tilde{O}(n^2 \log n)$ complexity. Other continuous techniques for computing geodesics, such as fast marching or solving the geodesic ODE, $\ddot{\gamma}^k + \Gamma^k_{ij}\dot{\gamma}^i\dot{\gamma}^j = 0$, exhibit similar $\tilde{O}(n^2)$ scaling for the all-pairs problem. For linear scaling, it is standard to pick $K \ll n$ landmark points, in which case one may instead compute a low-rank approximation to d^M, d^N with $\tilde{O}(n \log n \cdot K)$ complexity.

For (3), `MGW` uses the `ott-jax` solver Cuturi et al. (2022) to compute the optimal transport coupling for the Gromov-Wasserstein problem (2.3) using the Riemannian costs. The exact Gromov-Wasserstein problem is NP-hard Peyré et al. (2019), as similar geometric alignment problems in combinatorial pattern matching and imaging are also generally known to be NP-hard. However, Gromov-Wasserstein admits efficient approximations using entropic regularization (the Sinkhorn algorithm) with $\tilde{O}(n^2)$ iteration complexity Peyré et al. (2019). Furthermore, low-rank approximations Halmos et al. (2024) allow for $\tilde{O}(ndr)$ iteration complexity for rank r. Low-rank solvers are provided by the `ott-jax` library Cuturi et al. (2022) and are thus natively enabled for `MGW`.

We empirically profile the scalability of `MGW` in Figures S11, S12 for dimension $d = 30$, with 1000 training iterations of the neural field, and a range of sample complexities $N \in \{100, 200, 500, 1000, 2000, 3000, 4000, 5000\}$. `MGW` exhibits runtime scaling similar to that of spatial GW and FGW (Figure S11a), with a breakdown indicating that the geodesic computation scales quadratically like the Gromov-Wasserstein solver itself (Figure S11b, Figure S12).

On Hyperparameters and Consistency

In contrast to Fused Gromov-Wasserstein (FGW), `MGW` not require an explicit hyperparameter α to quantify the trade-off between and feature similarity and spatial similarity. The FGW objective function Vayer et al. (2020) for Euclidean feature cost c and spatial costs c_1 and c_2 Liu et al. (2023); Zeira et al. (2022); Klein et al. (2025) is a convex combination of the Wasserstein objective $\mathrm{C}_W(\boldsymbol{\pi}) = \sum_{ij} c_{ij}\boldsymbol{\pi}_{ij}$ (eqn. (7)) the and Gromov-Wasserstein objective $\mathrm{C}_{GW}(\boldsymbol{\pi}) = \sum \boldsymbol{\pi}_{ij}\boldsymbol{\pi}_{i'j'} \, ((c_1)_{ii'} - (c_2)_{jj'})^2$ (eqn. (2.3)) with hyperparameter $\alpha \in (0, 1)$:

$$\mathrm{FGW}_\alpha := \min_{\boldsymbol{\pi} \in \Pi_{a,b}} \alpha \cdot \mathrm{C}_W(\boldsymbol{\pi}) + (1 - \alpha) \cdot \mathrm{C}_{GW}(\boldsymbol{\pi}). \quad (13)$$

Hence, the underlying optimization problem FGW_α changes as a function of α with a different choice of α yielding a different weighting between modalities. Moreover the scalar α enforces a linear trade-off between dimensionally incompatible units (e.g., weighting physical microns against transcript counts), and thus the choice for α is ill-defined and often determined heuristically.

In comparison, `MGW` has a consistent optimization problem, with a loss function that does not depend on a hyperparameter to weight the contributions from different modalities. Specifically, the Jacobians $\boldsymbol{J}_\varphi$ and $\boldsymbol{J}_\psi$ act as local conversion operators, translating spatial displacement into the respective units of biological variation. Thus, `MGW` maps between different feature modalities $\mathcal{M} \neq \mathcal{N}$

in a dimensionally-consistent manner by leveraging the product structure of the Euclidean space E and the feature manifolds. `MGW` has other hyperparameters including the choice of k for computing geodesics and the parametrization of the neural fields $\psi_\eta, \varphi_\theta$, but importantly these are hyperparameters that govern the quality of the approximation of the objective function rather than the objective function itself. For instance, computing geodesics (6) with a fixed k and grid simply determines the resolution of discretization, while the neural fields $\psi_\eta, \varphi_\theta$ are trained to approximate the true underlying functions φ, ψ. Consequently, provided the geodesics (6) are accurately discretized, and the neural networks $\psi_\eta, \varphi_\theta$ converge to the underlying signals φ, ψ (in Sobolev norm, e.g. matching value and spatial derivative), `MGW` yields a unique, intrinsic solution.

Algorithm 1 Manifold GW (M-GW): Geodesic Distances via a pull-back metric on the common base E (Long Version)

Input : Samples
$\{(s^i, X^i)\}_{i=1}^n \subset E \times \mathcal{M}$,
$\{(t^j, Z^j)\}_{j=1}^m \subset E \times \mathcal{N}$;
Base metric g^E on E (e.g., Euclidean).
Output : Coupling $\pi^\star \in \Pi(\boldsymbol{a}, \boldsymbol{b})$ aligning the two datasets.

(A) Learn neural maps from base space E to feature spaces
Fit φ_θ by $\theta \leftarrow \arg\min_\theta \frac{1}{n}\sum_i \|\varphi_\theta(s^i) - X^i\|_2^2$;
Fit ψ_η by $\eta \leftarrow \arg\min_\eta \frac{1}{m}\sum_j \|\psi_\eta(t^j) - Z^j\|_2^2$.

(B) Build pull-back metrics on E
For each s^i: compute the Jacobian $J_\varphi(s^i) = D\varphi(s^i)$ (via automatic differentiation) and set

$$g^M(s^i) \ := \ J_\varphi(s^i)^\top J_\varphi(s^i).$$

For each t^j: compute the Jacobian $J_\psi(t^j) = D\psi(t^j)$ and set

$$g^N(t^j) \ := \ J_\psi(t^j)^\top J_\psi(t^j).$$

(C) Construct base-graphs on E with local arc-lengths.
On $\{s^i\}$ build k-NN graph $\mathcal{G}_M$. For edge (i,j) define $\Delta_{ij} = (s^i - s^j)$ and compute weights

$$w_M^{ij} \ = \ \frac{1}{2}\sqrt{\Delta_{ij}^\top g_i^M \Delta_{ij}} \ + \ \frac{1}{2}\sqrt{\Delta_{ij}^\top g_j^M \Delta_{ij}}.$$

Analogously build $\mathcal{G}_N$ on $\{t^j\}$ with edge lengths w_N^{jk} from g^N.

(D) Geodesic distances on E
Compute pairwise geodesic distances $d^M(s^i, s^{i'})$ on $\mathcal{G}_M$; $d^N(t^j, t^{j'})$ on $\mathcal{G}_N$.
Set $C^M_{ii'} = (d^M(s^i, s^{i'}))^2$, $C^N_{jj'} = (d^N(t^j, t^{j'}))^2$.

(E) Solve GW with pull-back geodesic costs
Given histograms $\boldsymbol{a} \in \Delta^n$, $\boldsymbol{b} \in \Delta^m$, solve

$$\pi^\star \in \arg\min_{\pi \in \Pi(\boldsymbol{a}, \boldsymbol{b})} \sum_{i,i',j,j'} (C^M_{ii'} - C^N_{jj'})^2 \, \pi_{ij} \, \pi_{i'j'}.$$

Experimental Details

Datasets, Availability, and Pre-processing.

In Section 3, we have evaluations of: (1) a spatiotemporal mouse transcriptomics Stereo-Seq dataset Chen et al. (2022) (Section 3.1), (2) an evaluation of a Visium 10x Genomics colorectal cancer (CRC) dataset and a Xenium dataset at the same slide Oliveira et al. (2025) (Section 3.2), (3) an alignment of a MALDI-MSI metabolomics dataset and Visium transcriptomics dataset of a human striatum brain section Vicari et al. (2023) (Section 3.4), and (4) and alignment of an AFADESI-MSI metabolomics and Visium transcriptomics dataset of human clear cell renal carcinoma (ccRCC) Tian et al. (2025) (Section 3.3). All datasets are publicly accessible, and we publish the code for loading and processing each.

Stereo-Seq Spatiotemporal Transcriptomics. For the dataset of Chen et al. (2022) we use `scanpy` to load AnnData files for each day of mouse embryonic development from E9.5 to E13.5

- `E9.5_E1S1.MOSTA.h5ad`
- `E10.5_E1S1.MOSTA.h5ad`
- `E11.5_E1S1.MOSTA.h5ad`
- `E12.5_E1S1.MOSTA.h5ad`
- `E13.5_E1S1.MOSTA.h5ad`

We construct paired datasets by taking the intersection of common genes across the timepoints, and perform a joint PCA across the time pairs. To do this, we concatenate each consecutive timepoint pair and normalize the joint AnnData with `sc.pp.normalize_total` and add pseudo-counts with `sc.pp.log1p` for feature-sparsity. We then perform PCA on the joint AnnData with 30 components. As the PCA is performed jointly in this experiment, no additional CCA step is required for feature-alignment. Owing to the size of the slices, we randomly downsample each to 10k points with a random seed of `rng`=42.

Visium-Xenium Alignment. For the public dataset of Oliveira et al. (2025) we use the colorectal cancer (CRC) dataset. This consists of two multi-modal sections from the same donor (Sample P2 CRC) Oliveira et al. (2025). This dataset includes a Visium CytAssist v2 section and a Xenium spatial transcriptomics section profiling the same tissue. We use this dataset, as it exhibits significant overlap ($\approx$ 90%, Zheng et al. (2025)), making it an appropriate candidate for alignment. As the Visium technology offers sequencing of the transcriptomics ($\approx 18,000$ genes), and Xenium offers FISH-based imagining (422 genes), we treat the datasets as distinct modalities. As a result, we perform independent PCA on each dataset (as opposed to a joint PCA) with 100 components. We then perform a CCA, as outlined in Section 10.8, with 40 components to find the most mutually correlated components within the independent PCA sets.

Transcriptomics-Metabolomics of Human Striatum. We download the Visium and MALDI-MSI datasets for the slide `V11T17-102_A1` from Vicari et al. (2023), corresponding to a post-mortem slice of human striatum in with Parkinson's diseases. The data deposition of Vicari et al. (2023) is available at Mendeley Data through the link https://data.mendeley.com/datasets/w7nw4km7xd/1. We select this slide in particular, as it is the only dataset with published annotations for (dopamine-annotated) Visium neurons which we use as a ground-truth in our benchmarking. These are contained in `dopamine.csv` in the directory `V11T17-102/V11T17-102_A1/output_data/V11T17-102_A1_RNA/outs/dopamine.csv`. We use the label `dopamine_Cd` as the ground-truth dopamine predictor in the caudate-nucleus (Cd), and use the labels `not_dopamine_Cd` and `CI` as the negative labels. We pre-process the metabolomics dataset with filtering for the top spatially-varying metabolites. We first normalize the metabolomics data with scanpy as `sc.pp.normalize_total`, and then use squidpy to compute the spatially variable metabolites with `sq.gr.spatial_neighbors` and `sq.gr.spatial_autocorr`, using `mode="moran"` to employ the Moran's I statistic. We select the top 20 metabolites in this step, and use this pre-processed data for all methods. For `MGW` we do not perform any additional processing of the raw metabolomics data beyond filtering. We use the CCA step (Section 10.8) to align the metabolomics and transcriptomics (PCA) features to 3 CC components in a joint-space suitable for `MGW` and use these features for alignment.

Transcriptomics-Metabolomics of Renal Cancer. To benchmark against the multimodal model `SpatialMeta`, we employed the clear cell renal cell carcinoma (ccRCC) dataset from its original publication Tian et al. (2025). The processed spatial transcriptomics (ST) and spatial metabolomics (SM) data for the `Y7_T` tissue section were obtained from the Zenodo repository (accession code 14986870), as `Y7_T` was the only slice for which both raw ST and SM data were publicly available. The corresponding ST-SM alignment generated by `SpatialMeta` was also downloaded from the same source.

For our method (`MGW`) and other optimal transport-based baselines, we followed `SpatialMeta`'s preprocessing workflow provided in their official GitHub repository (VHI_for_ST_SM). To ensure a fair comparison, we adopted the same variational autoencoder (VAE) architecture as `SpatialMeta` across all methods and trained each model for 100 epochs to obtain the low-dimensional embeddings. We repeat the evaluation of the entire across three random seeds to ensure reproducibility.

Model evaluation was performed using `SpatialMeta`'s official benchmarking pipeline. In particular, this was done via the function `run_modality_benchmark` which is implemented in `multi_benchmark_function.py` in the `benchmark/` folder. Since the manual pathological annotations referenced in the original publication were not included in the released dataset, metrics dependent on these annotations were excluded from our evaluation, and the remaining quantitative metrics were computed consistently across all models.

Baselines

We benchmark `MGW` against the methods `moscot` Klein et al. (2025), `SCOT` Demetci et al. (2022b), `SCOTv2` Demetci et al. (2022a), SpatialMeta Tian et al. (2025), and PASTE2 Liu et al. (2023), in addition to two baselines: Gromov-Wasserstein optimal transport on (1) features and (2) spatial coordinates. The feature input is standardized for all methods to the PCA components computed by `mgw.mgw_preprocess`. We run `moscot` `moscot.problems.TranslationProblem` with `max_iter=5_000` and `tol=1e-7`. Following the tutorial, available at moscot trans. tutorial, we use the default `alpha = 1` as one must ignore the `joint_attr` for multiomics alignment. For `SCOT` we following the procedure outlined in the tutorial SCOT tutorial. We set `k=50` for the nearest neighbor graph, `epsilon=0.005` for the entropic regularization, and set `normalize=True`. We use the same parameters for `SCOTv2`, with the additional parameter of `rho=0.1`. We run `PASTE2` with `s=0.7` and replace the `adata.X` coordinate with spatial coordinates, as multiomic alignment lacks joint distances across features. Lastly, for the Gromov-Wasserstein baselines, we use the package `ott-jax` Cuturi et al. (2022) with feature and spatial costs constructed with squared-Euclidean distance as $\|s^i - s^j\|_2^2$ and $\|t^i - t^j\|_2^2$ (spatial), as well as $\|X^i - X^j\|_2^2$ and $\|Z^i - Z^j\|_2^2$ (feature in spaces $\mathcal{M}$ and $\mathcal{N}$). We use the parameters `inner_maxit=3000`, `outer_maxit=3000`, `inner_tol=1e-8`, `outer_tol=1e-8`, `epsilon=1e-4` (Section 3.1), exactly paralleling the settings of `ott-jax` used for `MGW` but with a different cost. For the comparison to `SpatialMeta`, we downloaded their aligned `adata_joint_Y7_T_raw.h5ad` for `Y7_T` slice from their published Zenodo source https://zenodo.org/records/14986870. Then we trained the `MGW`, `SpatialMeta` and other models on

`ConditionalVAESTSM` from `SpatialMeta`'s Github for 100 epochs to retrieve the embedding for downstream analysis.

Ablation of MGW

We perform an ablation study to understand the source of performance in `MGW` Ȯn the mouse-embryo pairs in the dataset of Chen et al. (2022): `E9.5-E10.5`, `E10.5-E11.5`, `E11.5-E12.5`, and `E12.5-E13.5` we ablated (1) the effect of the spatial graph structure, (2) the effect of neural smoothing of the features, and (3) the effect of the neural Jacobian. In particular, we define an ablation study compare the following four regimes:

- **(1) Spatial Geodesics (Base Graph Structure).** Compute geodesics on spatial coordinates alone, using the identity metric on an unweighted k-NN graph.
- **(2) Feature GW on the Neural Field.** Train the neural field and use its smoothed output features to perform a Euclidean GW.
- **(3) Discrete finite-difference Pull-back Geodesics.** Compute geodesics on the spatial graph weighted by the discrete finite-difference approximation of the pull-back metric (i.e., the discrete approximation of local feature changes).
- **(4) Neural Pull-back Geodesics.** Complete `MGW`: extracting the continuous pull-back metric via the neural Jacobian to compute geodesics.

(1) Isolates whether constraining the transport to the spatial domain alone drives the improvement (i.e., isolating the effect of using the spatial boundaries of the slice), independent of features. (2) Isolates whether the gains come from neural filtering of high-frequency components of variation (i.e., noise), rather than the pull-back metric. (3) Isolates whether there is an advantage to using (gradient-based) continuous neural Jacobians, as opposed to a direct finite-difference approximation of the pull-back metric on the graph. The full MGW formulation (4) outperforms the isolated components (Figure S10). While Spatial Geodesics (1) achieve lower migration distance as expected (mean migration = 0.0850), the spatial boundary information alone is insufficient to improve biological alignment (AMI) relative to standard spatial GW with a lower mean AMI = 0.3236. Similarly, Neural Field (Smoothed) Feature GW exhibits a similar trend of under performance on migration (mean migration = 0.4658) as standard Feature GW exhibits, demonstrating that neural smoothing alone does not account for MGW's gain. This indicates the functional correspondence between physical space and feature space is required. Finally, while Discrete Finite-Difference Pull-back Geodesics (3) perform better than the base components (average migration = 0.1870, AMI = 0.3352), this finite-difference approximation remains sensitive to high-frequency technical noise. The Neural Pull-back Geodesics (4) are the most successful at capturing the true intrinsic geometry (average migration = 0.1201, AMI = 0.3617), and yield the optimal balance of biological feature alignment and spatial coherence of the respective ablations of MGW.

Sensitivity Analysis of k-NN Graph for Geodesics

To approximate continuous Riemannian geodesics, `MGW` relies on a discretization of the domain into a sparse neighborhood graph over the spatial coordinates. To assess sensitivity of `MGW` to this discretization step, we performed an ablation study on a size $N = 5000$ sub-sample of the `E9.5-E10.5` Stereo-seq time point pair of Chen et al. (2022). We (1) computed a k-NN graph for varying k and (2) investigated discretizing the graph with respect to neighbors in a fixed ϵ-ball for varying ϵ. We track both spatial coherence (migration) and feature alignment (AMI).

k-NN Sensitivity Analysis. We evaluated `MGW` across a range of neighborhood sizes $k \in \{6, 8, 10, 12, 14, 16, 18, 20, 22, 24\}$, holding the neural fields φ, ψ constant to isolate the effect of the graph topology on the geodesics. The neural fields converge to a loss of $0.00428, 0.00394$ after 20000 iterations. We find that, for an accurate learned representation of the two fields, `MGW` is robust to k (Table 5) with a migration in the range $\sim$ 9-10% and biological feature alignment AMI $\approx$0.38-0.42 both remaining stable for the range of k considered. This empirically validates the continuous geometric formulation: the performance is primarily driven by the continuous metric g^M, g^N inferred by the neural Jacobian, rather than the graph approximation parameter k. Our default choice for k in the experiments is $k = 12$. This reflects the local topology of the spatial grids (e.g., hexagonal arrays with 6 neighbors), ensuring robust connectivity beyond the first ring of neighborhoods while maintaining sparsity to ensure fast computation of the geodesics.

ϵ-Neighborhood Sensitivity Analysis. Next, we evaluate fixed radial fractions $r_{\text{frac}} := r \in \{0.01, 0.015, 0.02, 0.03\}$ of the largest diagonal in the spatial grid S as a means of generating neighborhoods for the graph. We find for $r \leq 0.01$ that the graph fractures into hundreds or thousands of disconnected components. This prevents global geodesic computation and causes the alignment to fail (Table 6). For viable radii ($r_{\text{frac}} \geq 0.015$), the method successfully aligns the data with $\sim$ 10% migration and AMI in range $\sim$0.38-0.40 (Table 6). However, unlike for the k-NN discretization which defines the exact number of neighbors, this approach requires tuning to avoid the graph becoming overly dense.

Architecture, Parameters, and Training of the Neural Fields.

To train our neural fields $\varphi : \mathbb{R}^2 \to \mathcal{M}$ and $\psi : \mathbb{R}^2 \to \mathcal{N}$, we use multi-layer perceptrons (MLPs). For ψ, φ to be smooth sub-manifolds with sufficiently smooth Jacobians we require smooth C^∞ activations: in particular, we exclude activation functions like ReLU and use the SiLU or SWISH function. We use an MLP with widths $(128, 256, 256, 128)$, learning rate $\eta = 1 \times 10^{-3}$, weight-decay of $w_{\text{decay}} = 1 \times 10^{-4}$, and EMA (exponential moving average) decay of $\text{EMA}_{\text{decay}} = 0.995$. We train the network for a total of `niter=20_000` training iterations. Because $\varphi : \mathbb{R}^2 \to \mathcal{M}$ and $\psi : \mathbb{R}^2 \to \mathcal{N}$ represent implicit neural fields (INF) over a contiguous spatial domain, their objective is to parametrize a single dataset rather than to generalize to unseen spatial coordinates. Consequently, accuracy is measured by the fidelity of recovering the observed signals, $X^i \approx \varphi_\theta(s^i)$, without a traditional train-test split (analogous to established INR approaches such as Sitzmann et al. (2020); Chitra et al. (2025)). In this context, "overfitting" would manifest as the memorization of high-frequency signals in the data such as technical noise. To prevent this, the INF acts as a low-pass filter by leveraging the spectral bias of MLPs. Combined with C^∞ smooth activations (SiLU) and early-stopping, this ensures the network captures true low-frequency spatial gradients while resisting high-frequency technical artifacts.

Computing metric tensor-field and geodesics.

We use `vmap(jacrev(phi))(X)` to compute Jacobians at each point, and compute the pull-back metric tensor field using an Einstein summation of the form `torch.einsum('nfd,nfe->nde', J, J)`. We normalize this tensor-field across space with the mean-eigenvalue of g as a proxy, and add a small stabilization of the form $g^M_\epsilon \leftarrow g^M + \epsilon \mathbf{I}$. As g^M is scaled ≈ 1 we typically choose $\epsilon = 10^{-2}$ to be much smaller, but sufficiently large to avoid instabilities for under-determined g.

To practically realize geodesics, we compute a K-nearest neighbor graph. On spatial positions $\{s^i\}$ we build k-NN graph $\mathcal{G}_M$. For edge (i,j) define $\Delta_{ij} = (s^i - s^j)$. Then, the edge-weights in $\mathcal{G}_M$ are given by the arc-lengths in the Riemannian metric

$$w_M^{ij} \;=\; \frac{1}{2}\sqrt{\Delta_{ij}^\top g_i^M \Delta_{ij}} \;+\; \frac{1}{2}\sqrt{\Delta_{ij}^\top g_j^M \Delta_{ij}}$$

With g^M defined from the neural field at each point $g^M(s^i)(\cdot,\cdot)$ and $g^M(s^j)(\cdot,\cdot)$. From this graph, geodesic distances are computed with Dykstra's algorithm from `scipy.sparse.csgraph.shortest_path`.

Details on the Optimal Transport.

For the optimal transport, we use the Gromov-Wasserstein solver of `ott-jax` Cuturi et al. (2022). This relies on using our instantiated geodesic costs as

- `geometry.Geometry(cost_matrix=CM)`
- `geometry.Geometry(cost_matrix=CN)`

and calling `quadratic_problem.QuadraticProblem` on these objects. We use parameters of `inner_maxit=3000`, `outer_maxit=3000`, `inner_tol=1e-8`, `outer_tol=1e-8`, and `epsilon=1e-4`. We offer the unbalanced setting with τ`_a` and τ`_b` as options, but set both to 1 (Balanced) for all experiments.

Pre-processing for the Alignment-Informative Feature Subset

Notably, Algorithm 1 for MGW takes the spaces $\mathcal{M}$, $\mathcal{N}$ as input and performs learning in step (i). However there are many ways to embed or represent each modality space $\mathcal{M}$, $\mathcal{N}$ as input to the algorithm in "step (0)." In particular, the feature spaces of distinct modalities need not align nor exhibit meaningful joint structure. There may be "marginal" sub-spaces of each feature which may be independent of the other modality, and there may be "joint" sub-spaces which exhibit high-correlation across the modalities. Thus, a natural pre-processing is to filter for such joint sub-spaces.

We propose a step based on *canonical correlation analysis* (CCA), which identifies linear combinations of two datasets which are maximally correlated. Formally, given random vectors $X \in \mathbb{R}^n, Y \in \mathbb{R}^m$ CCA computes a sequence of vectors $(a_k \in \mathbb{R}^n)_{k=1}^d, (b_k \in \mathbb{R}^m)_{k=1}^p$ that maximize the correlation of their projections $\rho = \mathrm{Corr}(a_k^\top X, b_k^\top Y)$. To isolate the joint sub-spaces, we propose an initial feature-refinement step comprising two components.

First, compute a coarse alignment $\hat{\boldsymbol{\pi}}$ between the two datasets $\mathbf{X}$ and $\mathbf{Z}$: an approximate correspondence is sufficient to identify features with strong cross-modal correspondence. *Second*, one takes a barycentric projection of the second dataset onto the first $\mathbf{Z}^\pi = \hat{\boldsymbol{\pi}}\,\mathrm{diag}(1/\hat{\boldsymbol{\pi}}^\top \mathbf{1}_n)\mathbf{Z}$. This ensures a 1-1 alignment of the points, which is a requirement of CCA. Following projection the canonical components are obtained by solving $\arg\max_{a_k,b_k} \mathrm{Corr}(a_k^\top \mathbf{X}, b_k^\top \mathbf{Z}^\pi)$ for $k \in [d]$. This yields canonical loading matrices $\mathbf{U}_a, \mathbf{U}_b$ with columns representing the maximally correlated directions of the two modalities. We then project the features in both modalities to their maximally-correlated components prior to `MGW` alignment. For a common modality, we perform a joint PCA across datasets in place of CCA.

Supplementary References

A. Chen et al. Spatiotemporal transcriptomic atlas of mouse organogenesis using DNA nanoball-patterned arrays. *Cell*, 185 (10):1777–1792, 2022.

U. Chitra et al. Mapping the topography of spatial gene expression with interpretable deep learning. *Nature Methods*, 22(2):298309, Jan. 2025.

M. Cuturi et al. Optimal Transport Tools (OTT): A JAX Toolbox for all things Wasserstein. *arXiv preprint arXiv:2201.12324*, 2022.

P. Demetci et al. Scotv2: Single-cell multiomic alignment with disproportionate cell-type representation. *Journal of Computational Biology*, 29:12131228, Nov. 2022a.

P. Demetci et al. Scot: single-cell multi-omics alignment with optimal transport. *Journal of Computational Biology*, 29(1):3–18, 2022b.

P. Halmos et al. Low-Rank Optimal Transport through Factor Relaxation with Latent Coupling. In *The Thirty-eighth Annual Conference on Neural Information Processing Systems*, 2024.

D. Klein et al. Mapping cells through time and space with moscot. *Nature*, Jan. 2025.

X. Liu, R. Zeira, and B. J. Raphael. Partial alignment of multislice spatially resolved transcriptomics data. *Genome Research*, 33(7): 1124–1132, 2023.

F. Mémoli. Gromov–Wasserstein Distances and the Metric Approach to Object Matching. *Foundations of computational mathematics*, 11:417–487, 2011.

M. F. d. Oliveira et al. High-definition spatial transcriptomic profiling of immune cell populations in colorectal cancer. *Nature Genetics*, 57(6):15121523, June 2025.

G. Peyré et al. Computational optimal transport: With applications to data science. *Foundations and Trends® in Machine Learning*, 11(5-6):355–607, 2019.

V. Sitzmann et al. Implicit neural representations with periodic activation functions. In *NeurIPS*, NIPS '20, Red Hook, NY, USA, 2020. Curran Associates Inc. ISBN 9781713829546.

R. Tian et al. Integrating cross-sample and cross-modal data for spatial transcriptomics and metabolomics with spatialmeta. *Nature Communications*, 16(1), Oct. 2025.

T. Vayer et al. Fused Gromov-Wasserstein distance for structured objects. *Algorithms*, 13(9):212, Aug. 2020.

M. Vicari et al. Spatial multimodal analysis of transcriptomes and metabolomes in tissues. *Nat. Biotech.*, 42(7):10461050, Sept. 2023.

R. Zeira et al. Alignment and integration of spatial transcriptomics data. *Nature Methods*, 19(5):567–575, 2022.

H. Zheng et al. Joint imputation and deconvolution of gene expression across spatial transcriptomics platforms. Feb. 2025.

Supplementary Figures and Tables

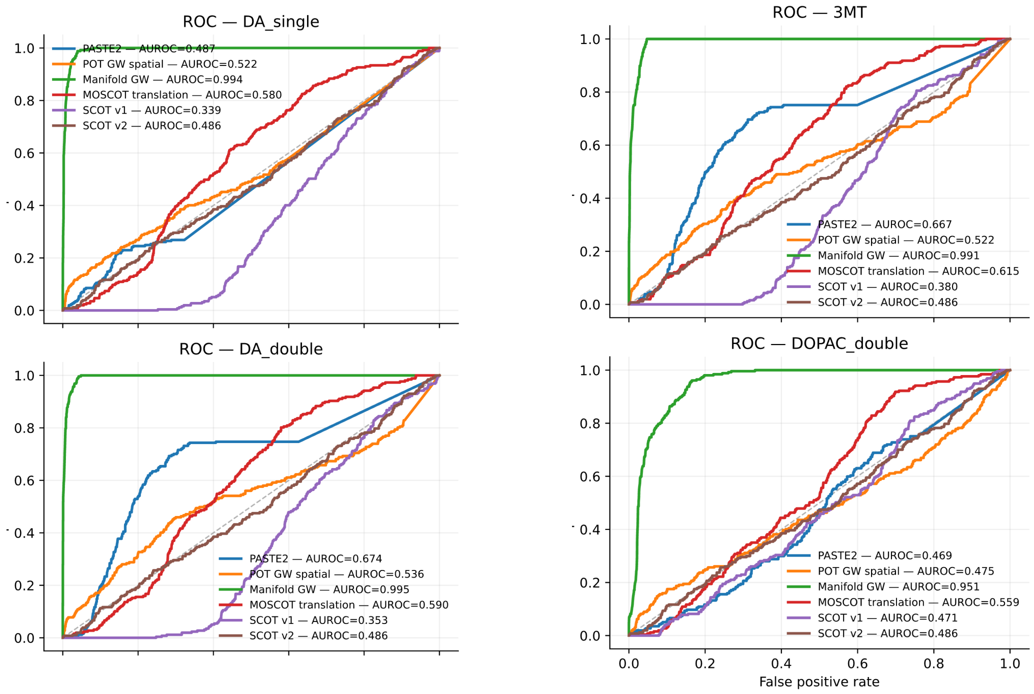

Fig. S7. AUROC curves for the 4 dopamine metabolites DA Single (singly-derivatized $m/z = 421.19$), DA double (doubly-derivatized $m/z = 674.28$), DOPAC double (dopamine-breakdown product $m/z = 698.24$), and 3MT (dopamine-breakdown product 3-Methoxytyramine, $m/z = 435.21$) in the MALDI-MSI to Visium Transfer Task.

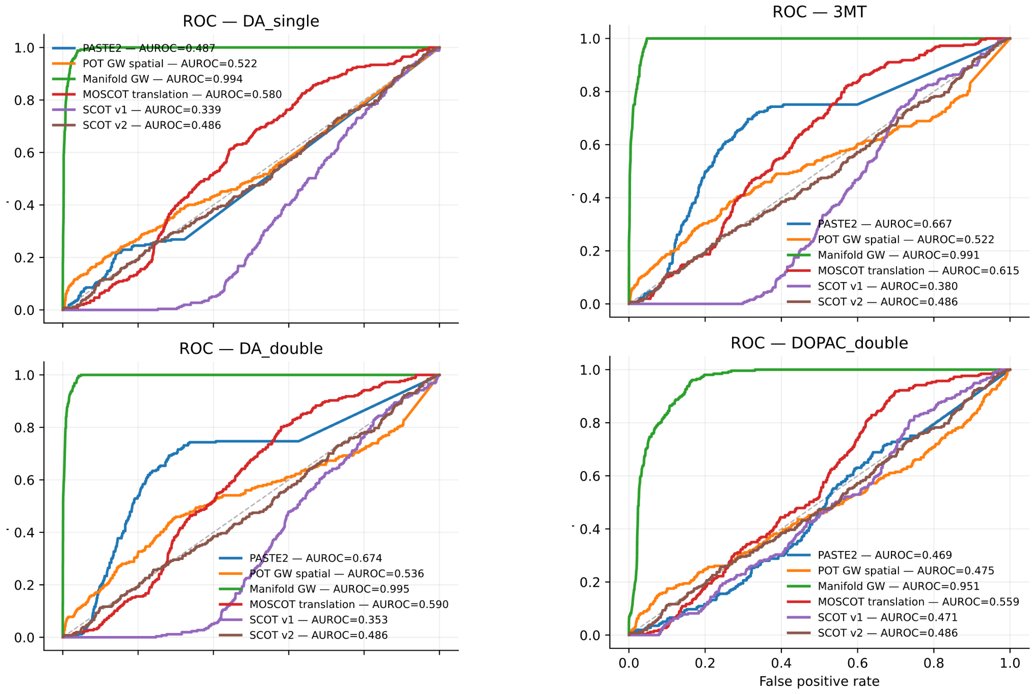

Fig. S8. AUPRC curves for the 4 dopamine metabolites DA Single (singly-derivatized $m/z = 421.19$), DA double (doubly-derivatized $m/z = 674.28$), DOPAC double (dopamine-breakdown product $m/z = 698.24$), and 3MT (dopamine-breakdown product 3-Methoxytyramine, $m/z = 435.21$) in the MALDI-MSI to Visium Transfer Task.

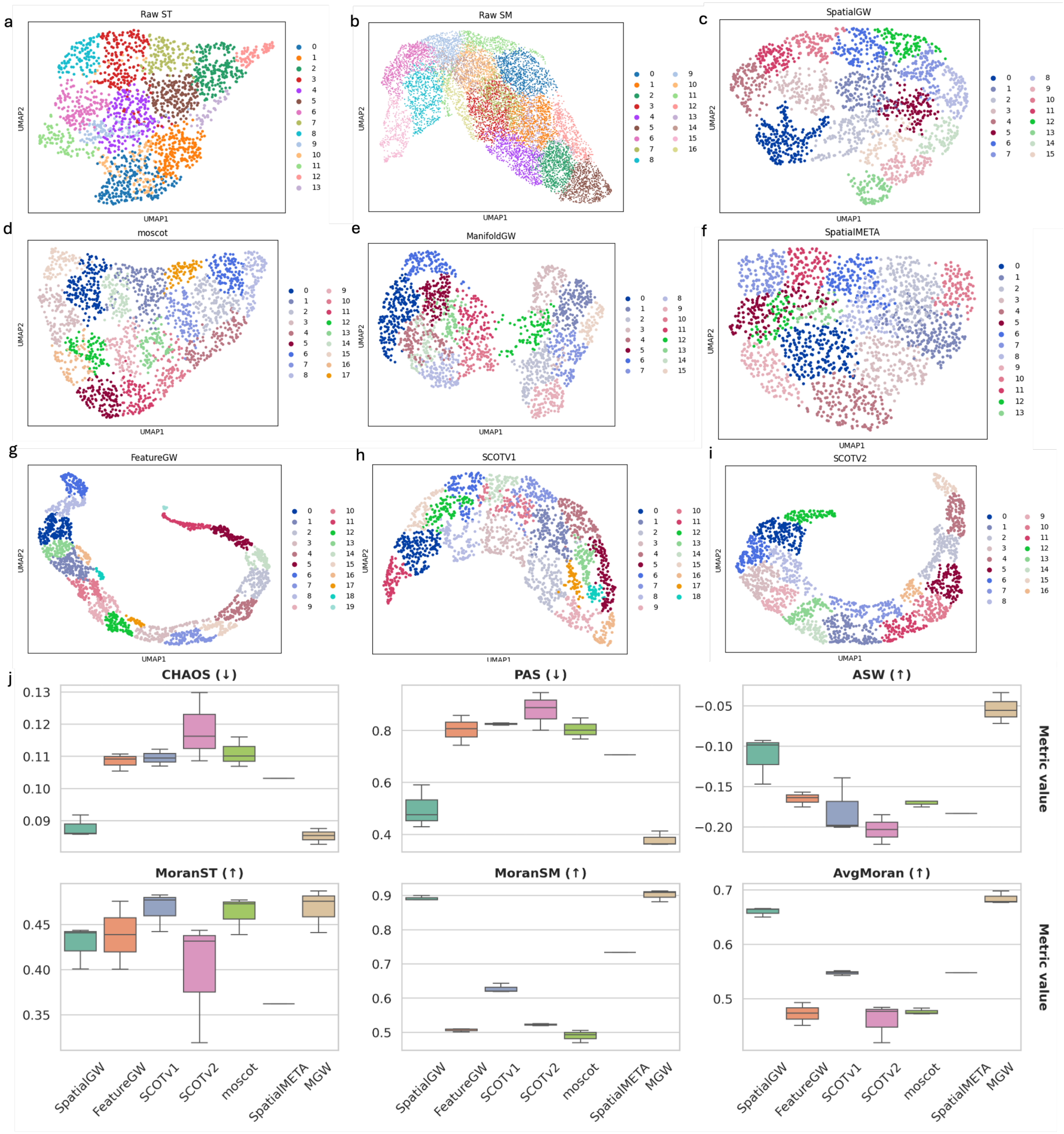

Fig. S9. (a-i) The UMAP visualization of the aligned embeddings of Y_T slice from ccRCC dataset of all models.**(j)** The boxplot of all six metrics across three seeds as in table 1. The `SpatialMeta`'s value is consistent across three seeds, because it is not an optimal transport based model and there is no randomness coming from the approximation of coupling Tian et al. (2025). The up or down arrow next to the metric stands for if a higher or lower value means better performance.

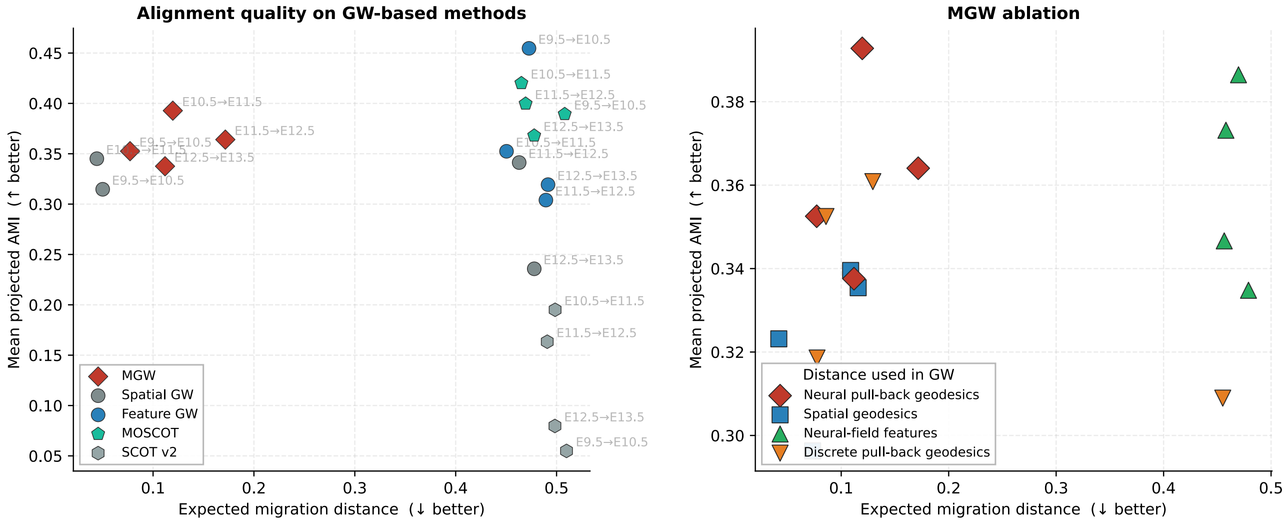

Fig. S10. Ablation Study of the components of `MGW` plotting in **(a)** the original performance against baselines as a reference, and **(b)** the ablation components of Section 10.3: Spatial Geodesics (Base Graph Structure), Feature GW on the Neural Field, Discrete finite-difference Pull-back Geodesics, and Neural Pull-back Geodesics (`MGW`).

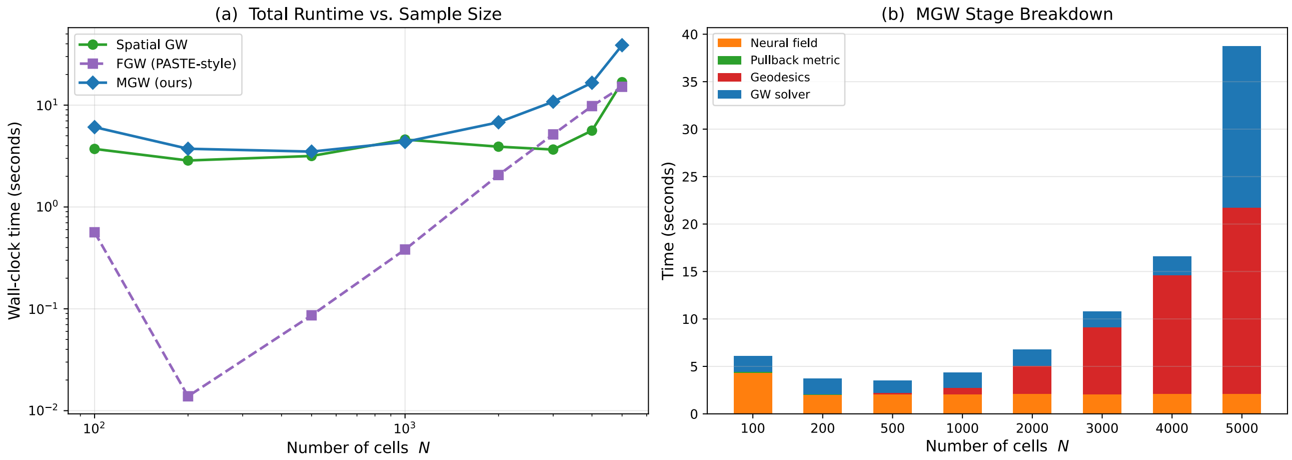

Fig. S11. Runtime of `MGW` on a Synthetic $d = 30$ dataset across sample size N with **(a)** Comparison of `MGW` GW, FGW, **(b)** Profiling of the `MGW` sub-routines (1) neural field training (1000 fixed iterations), (2) computation of the pull-back metric g^M, g^N, (3) computation of geodesic distances d_M, d_N, and (4) time spent solving the Gromov-Wasserstein problem with respect to d_M, d_N.

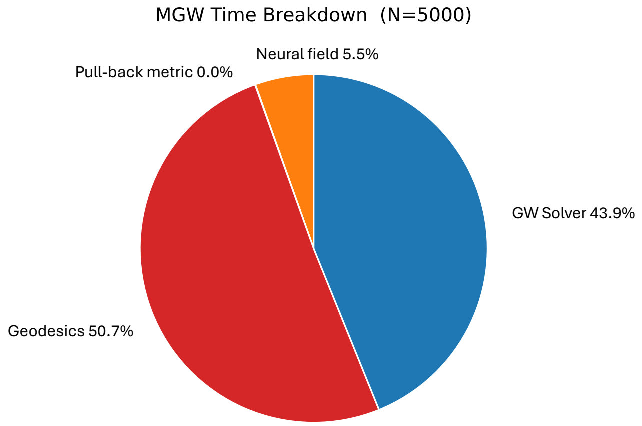

Fig. S12. Runtime profiling for $N = 6000$ of MGW on Synthetic $d = 30$ dataset in terms of cost for (1) neural field training (1000 fixed iterations), (2) pull-back metric, (3) geodesics distances, and (4) solving the Gromov-Wasserstein problem.

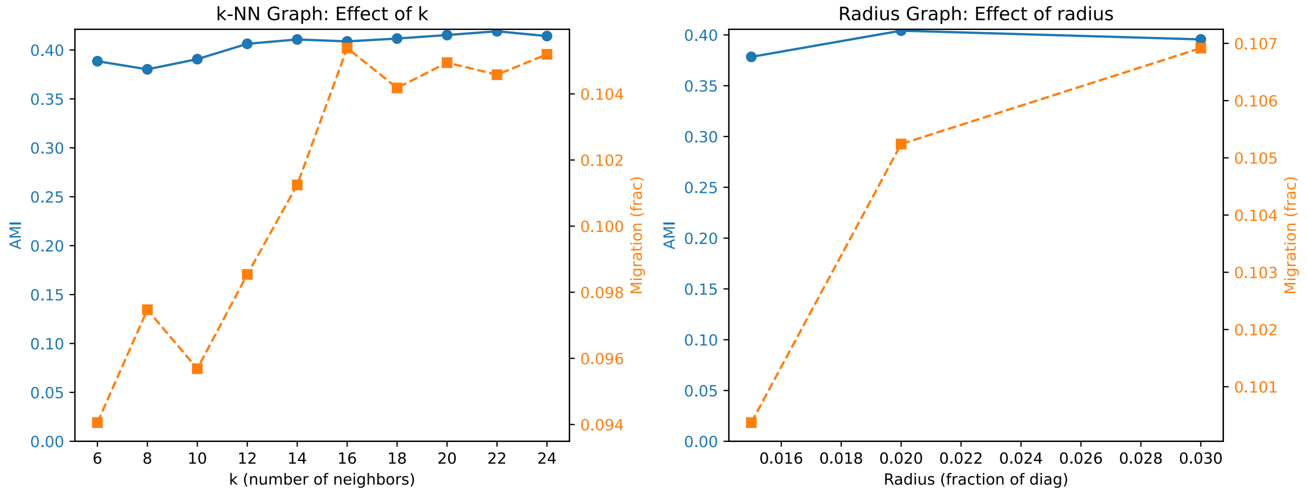

Fig. S13. (Left) Sensitivity analysis of MGW across a range of k-NN parameters k for the E9.5-E10.5 Stereo-seq pair Chen et al. (2022) (sub-sampled to $N = 5000$) in terms of spatial coherence (Migration) and biological accuracy (AMI). Alignment metrics remain stable across a wide range of k. **(Right)** Sensitivity analysis of MGW for an alternative ϵ-neighborhood-based discretization across a range of radii r, expressed as a fraction of the spatial domain's diagonal.

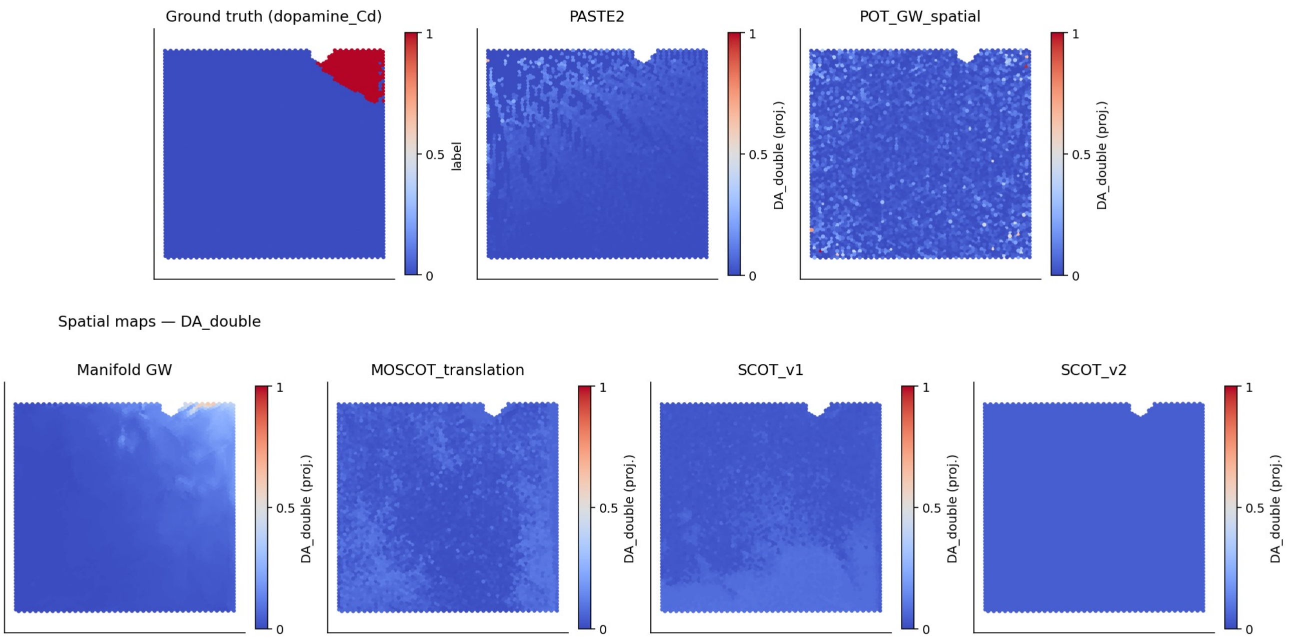

Fig. S14. Raw, unscaled DA Double (doubly-derivatized dopamine, $m/z = 674.28$) following barycentric projection of metabolite intensities onto Visium Slide of Vicari et al. (2023) across couplings π returned by various methods.

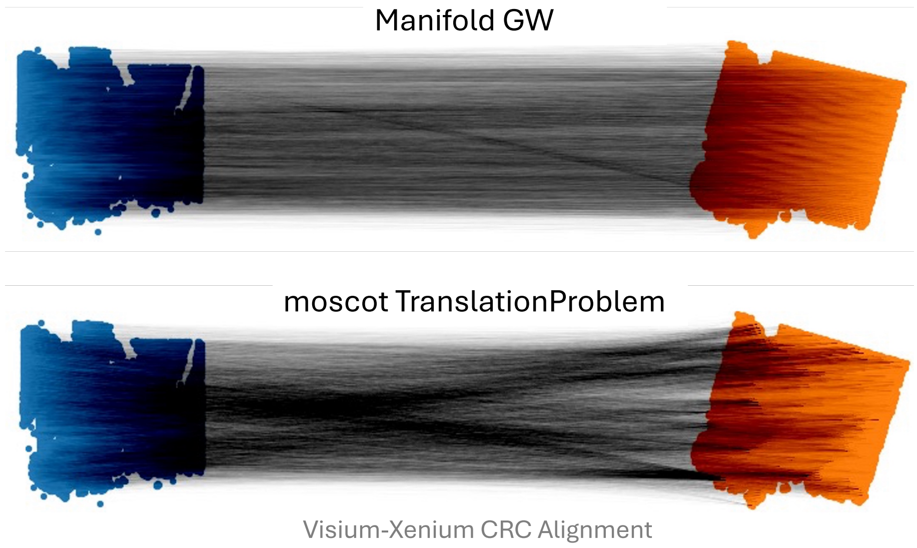

Fig. S15. Comparison on Visium and Xenium datasets of colorectal cancer Oliveira et al. (2025). Visualization of `MGW` and `moscot TranslationProblem` alignment.

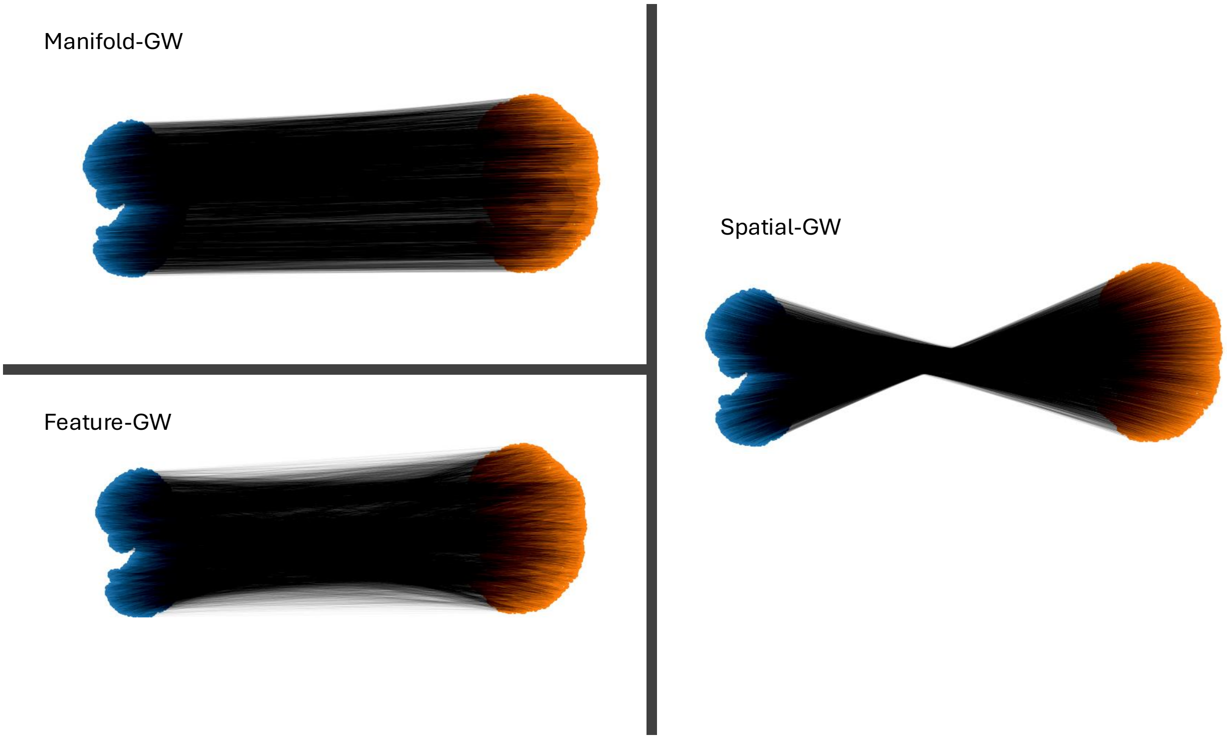

Fig. S16. Visualization of alignments computed by Manifold-GW, as well as Gromov-Wasserstein baselines Spatial-only GW and Feature-only GW. Alignments shown for Stereo-seq mouse-embryo timepairs E12.5-13.5 from Chen et al. (2022). In this pair, spatial GW exhibits a symmetry-breaking failure mode in aligning multi-modal data using spatial information alone.

Table 1. Comparison of alignment methods across metrics across three random seeds. Best scores per metric are shown in bold.

Method	CHAOS	PAS	ASW	Moran's I (ST)	Moran's I (SM)	Avg Moran's I
SpatialGW	0.0918	0.5906	-0.0984	0.4007	0.8998	0.6502
FeatureGW	0.1092	0.8582	-0.1568	0.4389	0.5091	0.4740
SCOTv1	0.1095	0.8290	-0.2004	0.4771	0.6193	0.5482
SCOTv2	0.1298	0.9459	-0.2033	0.3194	0.5198	0.4196
moscot	0.1101	0.8012	-0.1682	0.4389	0.5061	0.4725
SpatialMETA	0.1032	0.7063	-0.1834	0.3623	0.7334	0.5478
MGW	**0.0876**	**0.4127**	**-0.0717**	**0.4869**	**0.9090**	**0.6979**
SpatialGW	0.0858	0.4757	-0.1469	0.4406	0.8880	0.6643
FeatureGW	0.1054	0.7433	-0.1636	0.4006	0.5017	0.4511
SCOTv1	0.1070	0.8211	-0.1391	0.4421	0.6435	0.5428
SCOTv2	0.1086	0.8012	-0.1849	0.4436	0.5255	0.4845
moscot	0.1069	0.7675	-0.1752	**0.4728**	0.4931	0.4829
SpatialMETA	0.1032	0.7063	-0.1834	0.3623	0.7334	0.5478
MGW	**0.0854**	**0.3642**	**-0.0556**	0.4410	**0.9130**	**0.6770**
SpatialGW	0.0861	0.4291	-0.0928	0.4436	**0.8878**	0.6657
FeatureGW	0.1107	0.8072	-0.1752	0.4758	0.5102	0.4930
SCOTv1	0.1122	0.8265	-0.1978	**0.4826**	0.6209	0.5517
SCOTv2	0.1163	0.8880	-0.2214	0.4315	0.5224	0.4769
moscot	0.1160	0.8488	-0.1681	0.4771	0.4701	0.4736
SpatialMETA	0.1032	0.7063	-0.1834	0.3623	0.7334	0.5478
MGW	**0.0827**	**0.3622**	**-0.0336**	0.4758	0.8815	**0.6786**
**Average**						
SpatialGW	0.0879	0.4985	-0.1127	0.4283	0.8919	0.6601
FeatureGW	0.1084	0.8029	-0.1652	0.4384	0.5070	0.4727
SCOTv1	0.1096	0.8255	-0.1791	0.4673	0.6279	0.5476
SCOTv2	0.1182	0.8784	-0.2032	0.3982	0.5226	0.4603
moscot	0.1110	0.8058	-0.1705	0.4629	0.4898	0.4763
SpatialMETA	0.1032	0.7063	-0.1834	0.3623	0.7334	0.5478
MGW	**0.0852**	**0.3797**	**-0.0536**	**0.4679**	**0.9012**	**0.6845**

Table 2. Pairwise alignment performance across developmental stages. Lower migration and higher projected AMI indicate better cross-slice alignment quality.

Pair	Method	Migration ↓	Proj. AMI (A↔B) ↑
E9.5→E10.5	FeatureGW	0.473	0.455
E9.5→E10.5	MGW	0.077	0.353
E9.5→E10.5	MOSCOT_translation	0.508	0.389
E9.5→E10.5	SCOT_v2	0.510	0.055
E9.5→E10.5	SpatialGW	0.050	0.315
E10.5→E11.5	FeatureGW	0.450	0.352
E10.5→E11.5	MGW	0.120	0.393
E10.5→E11.5	MOSCOT_translation	0.465	0.420
E10.5→E11.5	SCOT_v2	0.499	0.195
E10.5→E11.5	SpatialGW	0.044	0.345
E11.5→E12.5	FeatureGW	0.489	0.304
E11.5→E12.5	MGW	0.172	0.364
E11.5→E12.5	MOSCOT_translation	0.469	0.400
E11.5→E12.5	SCOT_v2	0.491	0.163
E11.5→E12.5	SpatialGW	0.463	0.341
E12.5→E13.5	FeatureGW	0.492	0.319
E12.5→E13.5	MGW	0.112	0.338
E12.5→E13.5	MOSCOT_translation	0.478	0.368
E12.5→E13.5	SCOT_v2	0.498	0.080
E12.5→E13.5	SpatialGW	0.478	0.236

Table 3. Comparison of alignment methods on cosine similarity and migration metrics for Visium-Xenium alignment (CRC Colorectal Cancer tumor sample).

Method	Mean Cosine	Median Cosine	Migration
`MGW`	0.671	0.689	0.014
Spatial GW	0.660	0.681	0.218
Feature GW	0.744	0.753	0.239
Moscot	0.742	0.754	0.234
SCOT	0.665	0.694	0.234
SCOTv2	0.714	0.725	0.248
PASTE	0.437	0.455	0.001
POT	0.722	0.738	0.278

Table 4. Comparison of alignment methods across dopamine-related MSI targets. Best scores per target are shown in bold.

Method	Target	m/z	AUROC	AUPRC
Manifold GW	3MT	435.21	**0.991**	**0.846**
PASTE2	3MT	435.21	0.667	0.084
MOSCOT (translation)	3MT	435.21	0.615	0.063
POT–GW (spatial)	3MT	435.21	0.522	0.081
SCOT_v2	3MT	435.21	0.486	0.051
SCOT_v1	3MT	435.21	0.380	0.038
Manifold GW	DA_double	674.28	**0.995**	**0.907**
PASTE2	DA_double	674.28	0.674	0.090
MOSCOT (translation)	DA_double	674.28	0.590	0.058
POT–GW (spatial)	DA_double	674.28	0.536	0.085
SCOT_v2	DA_double	674.28	0.486	0.051
SCOT_v1	DA_double	674.28	0.353	0.037
Manifold GW	DA_single	421.19	**0.994**	**0.882**
MOSCOT (translation)	DA_single	421.19	0.580	0.057
POT–GW (spatial)	DA_single	421.19	0.522	0.081
PASTE2	DA_single	421.19	0.487	0.055
SCOT_v2	DA_single	421.19	0.486	0.051
SCOT_v1	DA_single	421.19	0.339	0.036
Manifold GW	DOPAC_double	698.24	**0.951**	**0.455**
MOSCOT (translation)	DOPAC_double	698.24	0.559	0.054
SCOT_v2	DOPAC_double	698.24	0.486	0.051
POT–GW (spatial)	DOPAC_double	698.24	0.475	0.061
SCOT_v1	DOPAC_double	698.24	0.471	0.045
PASTE2	DOPAC_double	698.24	0.469	0.046

Table 5. Sensitivity analysis of the k-NN graph parameter k for geodesic approximation on the E9.5 $\rightarrow$ E10.5 Stereo-Seq mouse embryo alignment sub-sampled to $N = 5000$ points.

Neighbors (k)	AMI ↑	Migration ↓	Runtime (s)
6	0.3885	**0.0941**	37.5
8	0.3802	0.0975	**28.7**
10	0.3907	0.0957	43.4
12	0.4063	0.0985	34.5
14	0.4108	0.1012	35.1
16	0.4088	0.1054	46.8
18	0.4117	0.1042	63.9
20	0.4153	0.1049	51.3
22	**0.4191**	0.1046	55.2
24	0.4143	0.1052	54.0

Table 6. Performance and computational cost of fixed-radius (ϵ-ball) graph construction on the E9.5 $\rightarrow$ E10.5 mouse embryo alignment. Radii are expressed as a fraction of the spatial domain's diagonal.

Radius Fraction	AMI ↑	Migration ↓	Runtime (s)
0.010	*Failed (Graph fractured into 164 components)*		
0.015	0.3784	**0.1004**	**34.0**
0.020	**0.4042**	0.1052	37.6
0.030	0.3956	0.1069	49.2

Table 7. Ablation of the `MGW` geometric stack across spatiotemporal Stereo-seq mouse embryo pairs. Migration represents the required spatial displacement as a fraction of the slide (lower is better), while AMI indicates biological feature alignment accuracy (higher is better).

**Time Pair**	**Method**	**Migration** (↓)	**AMI** (↑)
E9.5 → E10.5	Spatial Geodesics	0.0734	0.2963
	Neural Field Feature GW	0.4697	0.3865
	Discrete Pull-back Geodesics	0.0775	0.3186
	`MGW` (Neural Pull-back)	0.0772	0.3525
E10.5 → E11.5	Spatial Geodesics	0.1088	0.3395
	Neural Field Feature GW	0.4580	0.3732
	Discrete Pull-back Geodesics	0.4551	0.3089
	`MGW` (Neural Pull-back)	0.1196	0.3928
E11.5 → E12.5	Spatial Geodesics	0.1158	0.3354
	Neural Field Feature GW	0.4564	0.3466
	Discrete Pull-back Geodesics	0.1294	0.3608
	`MGW` (Neural Pull-back)	0.1716	0.3641
E12.5 → E13.5	Spatial Geodesics	0.0419	0.3232
	Neural Field Feature GW	0.4790	0.3348
	Discrete Pull-back Geodesics	0.0858	0.3525
	`MGW` (Neural Pull-back)	0.1119	0.3376
